# Tris[(1-isopropylbenzimidazol-2-yl)dimethylsilyl]methyl metal complexes, [Tism^Pr^i^Benz^]M: a new class of metallacarbatranes, isomerization to a tris(N-heterocyclic carbene) derivative, and evidence for an inverted ligand field†
†Electronic supplementary information (ESI) available: Experimental details. CCDC 1529724–1529731. For ESI and crystallographic data in CIF or other electronic format see DOI: 10.1039/c7sc00499k


**DOI:** 10.1039/c7sc00499k

**Published:** 2017-05-02

**Authors:** Serge Ruccolo, Michael Rauch, Gerard Parkin

**Affiliations:** a Department of Chemistry , Columbia University , New York 10027 , USA . Email: parkin@columbia.edu

## Abstract

The tris[(1-isopropylbenzimidazol-2-yl)dimethylsilyl]methyl ligand, [Tism^Pr^i^Benz^], has been employed to form carbatrane compounds of both the main group metals and transition metals, namely [Tism^Pr^i^Benz^]Li, [Tism^Pr^i^Benz^]MgMe, [Tism^Pr^i^Benz^]Cu and [Tism^Pr^i^Benz^]NiBr. In addition to the formation of atranes, a zinc compound that exhibits κ^3^-coordination, namely [κ^3^-Tism^Pr^i^Benz^]ZnMe, has also been obtained. Furthermore, the [Tism^Pr^i^Benz^] ligand may undergo a thermally induced rearrangement to afford a novel tripodal tris(N-heterocyclic carbene) variant, as shown by the conversion of [Tism^Pr^i^Benz^]Cu to [κ^4^-*C*_4_-Tism^Pr^i^Benz*^]Cu. The transannular M–C bond lengths in the atrane compounds are 0.19–0.32 Å longer than the sum of the respective covalent radii, which is consistent with a bonding description that features a formally zwitterionic component. Interestingly, computational studies demonstrate that the Cu–C_atrane_ interactions in [Tism^Pr^i^Benz^]Cu and [κ^4^-*C*_4_-Tism^Pr^i^Benz*^]Cu are characterized by an “inverted ligand field”, in which the occupied antibonding orbitals are localized more on carbon than on copper.

## Abstract 2

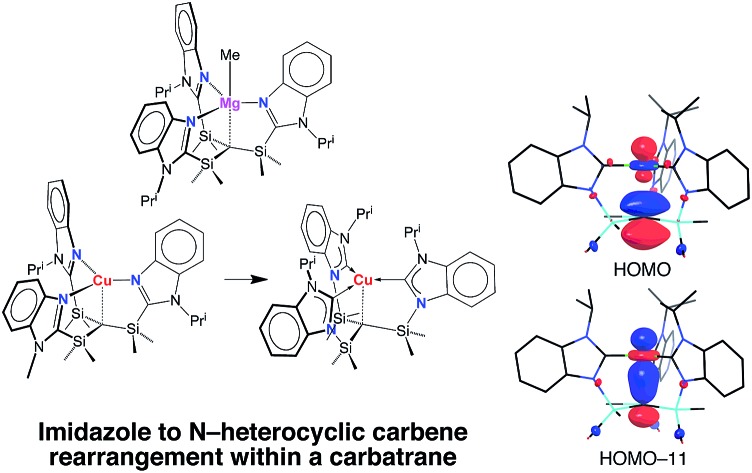
The tris[(1-isopropylbenzimidazol-2-yl)dimethylsilyl]methyl ligand, [Tism^Pr^i^Benz^], has been employed to form carbatrane compounds of both the main group metals and transition metals.

## Introduction

**Fig. 1:**
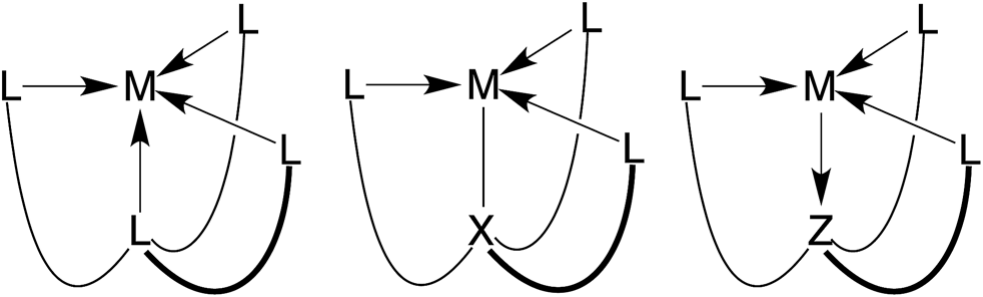
Three types of atrane molecules classified according to whether the transannular interaction involves an L, X or Z-type binding site.

Atranes comprise an interesting class of molecules in which two bridgehead atoms are joined by three three-atom linkers, thereby resulting in a tricyclic motif.^1^–^3^ A relatively recent development in this area is concerned with the synthesis of metallacarbatranes that feature transannular M–C interactions.^4^ Such compounds are of interest because the M–C bond corresponds to an M–X interaction, in contrast to the transannular M←L^4^,^5^ or M→Z^4^,^6^ dative bonds that are more commonly encountered in atranes (Fig. 1).^7^,^8^ For example, we have recently employed tris(2-pyridylthio)methyl ([Tptm])^3^,^9^ and tris(1-methylimidazol-2-ylthio)methyl ([Titm^Me^])^10^ as ligands for the construction of metallacarbatranes,^11^ and have demonstrated that the nature of the heterocyclic nitrogen donor has an impact on the structure of the carbatrane.^10^ Since a common feature of these ligands is the attachment of the heterocycles to the carbon bridgehead *via* a sulfur atom, we considered it worthwhile to investigate a different type of linker. Therefore, we report here a new class of tetradentate tripodal ligands in which three imidazole donors are attached to a carbon bridgehead *via* C–Si linkages. In addition, we also describe isomerization of the tris(imidazole) ligand to afford a novel tripodal tris(N-heterocyclic carbene) derivative.

## Results and discussion

We considered the [Me_2_Si] moiety to be an appealing linker for the construction of analogues of the above tetradentate tripodal ligands because (i) C–Si bonds are typically robust,^12^ (ii) methyl substituents on silicon can provide a protective environment for the bridgehead carbon, and (iii) silyl groups lower the p*K*_a_ of adjacent C–H groups,^13^ thereby facilitating protolytic cleavage. Furthermore, tripodal molecules of the type HC(SiMe_2_X)_3_ are known, *e.g.* X = NR,^14^ PR_2_,^15^ CH_2_PR_2_,^16^ S,^17^ Se,^17^ and OC_2_H_4_OMe,^18^ and thus provide a precedent for the synthesis of variants that include heterocyclic nitrogen donors.

**Scheme 1:**
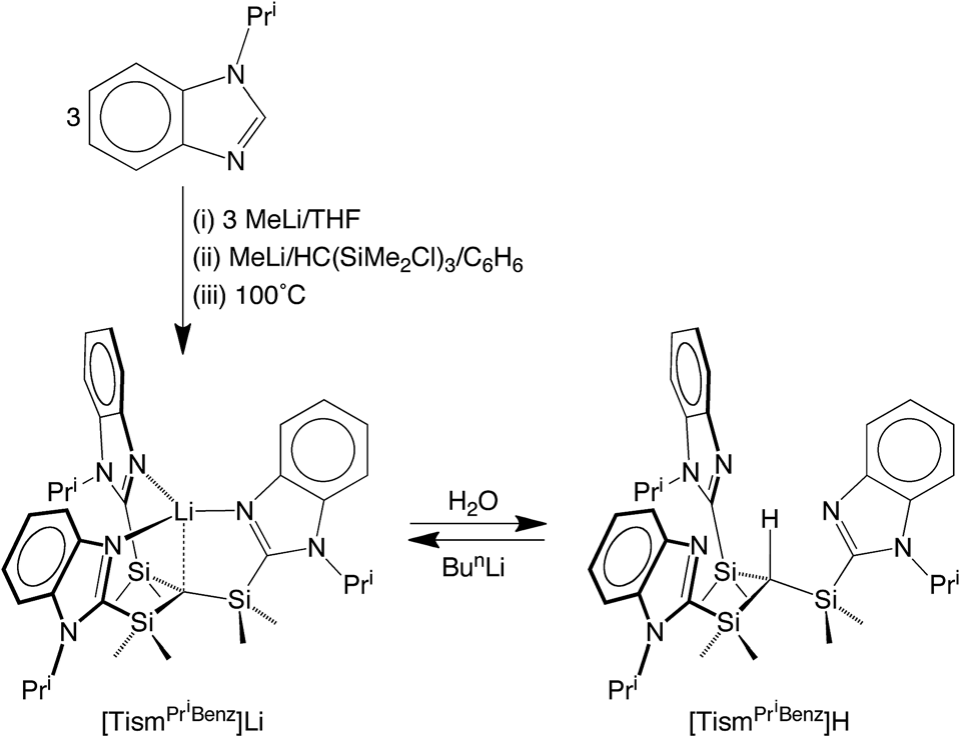


Indeed, tris[(1-isopropylbenzimidazol-2-yl)dimethylsilyl]-methane, [Tism^Pr^i^Benz^]H, and the lithium derivative, [Tism^Pr^i^Benz^]Li, may be obtained from 1-isopropylbenzimidazole and HC(SiMe_2_Cl)_3_*via* the sequence illustrated in Scheme 1.^19^ Specifically, treatment of 1-isopropylbenzimidazole with MeLi, followed by addition of HC(SiMe_2_Cl)_3_, affords [Tism^Pr^i^Benz^]Li, which is converted to [Tism^Pr^i^Benz^]H upon reaction with H_2_O;^20^ [Tism^Pr^i^Benz^]Li may also be regenerated by treatment of [Tism^Pr^i^Benz^]H with Bu^*n*^Li.

**Fig. 2:**
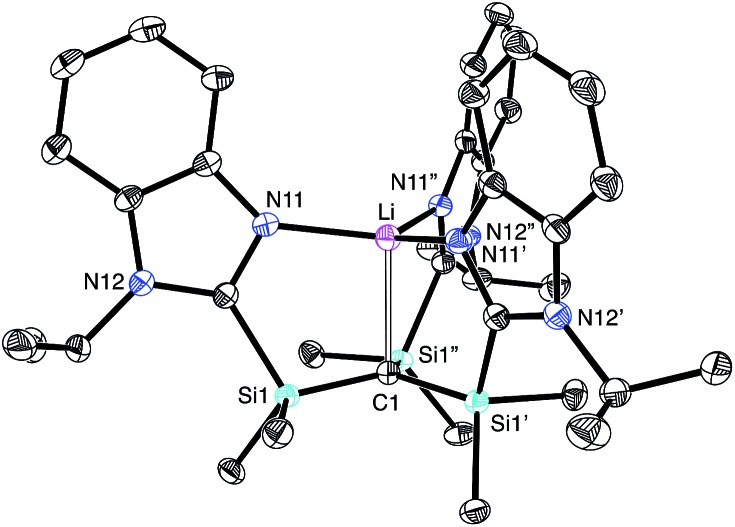
Molecular structure of [Tism^Pr^i^Benz^]Li.

**Fig. 3:**
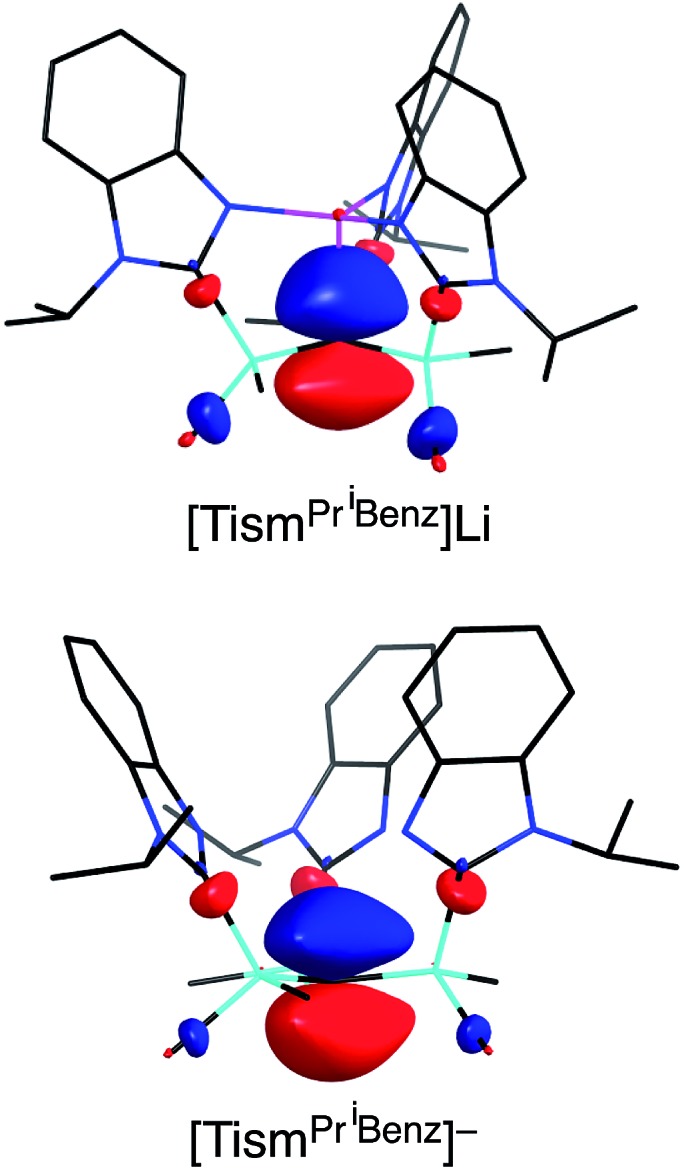
Comparison of the HOMO of [Tism^Pr^i^Benz^]Li (top) and [Tism^Pr^i^Benz^]^–^ (bottom).

The molecular structure of [Tism^Pr^i^Benz^]Li has been determined by X-ray diffraction (Fig. 2), thereby revealing that the compound possesses an atrane motif^21^ in which the lithium adopts an approximately trigonal monopyramidal^22^ coordination environment with N–Li–N and C–Li–N bond angles of 118.61(9)° and 96.8(2)°.^23^,^24^ Trigonal monopyramidal coordination is not common for lithium, but a similar coordination environment is observed for tris(2-pyridylthio)methyllithium, [Tptm]Li.^3^,^25^ An interesting difference between [Tism^Pr^i^Benz^]Li and [Tptm]Li, however, pertains to the geometry at the bridgehead carbon atom. Specifically, the [CSi_3_] moiety of [Tism^Pr^i^Benz^]Li adopts a much greater degree of planarity (Table 1) than does the [CS_3_] moiety of [Tptm]Li, as indicated by the fact that the sum of the Si–C–Si angles of [Tism^Pr^i^Benz^]Li (355.8°)^26^ is much closer to 360° than is the sum of the S–C–S angles of [Tptm]Li (345.2°). Furthermore, the Li–C–Si angles of [Tism^Pr^i^Benz^]Li [96.87(13)°] are closer to 90° than are the Li–C–S angles of [Tptm]Li [103.05(7)°].^27^,^28^ Since silyl-substituted carbanions are close to planar,^29^ as illustrated by [Li(tmen)_2_][C(SiMe_2_PPh_2_)_3_],^30^,^31^,^32^ the planarity of the [CSi_3_] moiety of [Tism^Pr^i^Benz^]Li suggests that the molecule possesses a significant degree of formally zwitterionic character in which carbon bears a negative charge.^4^,^33^–^35^ In support of this suggestion, while the Li–N bond lengths [2.017(2) Å] are comparable to the sum of the covalent radii (1.99 Å),^36^ the Li–C bond length [2.273(9) Å] is distinctly longer (by 0.23 Å) than the sum of covalent radii (2.04 Å).^36^,^37^ Moreover, the HOMO of [Tism^Pr^i^Benz^]Li is largely composed of a p-orbital on carbon, similar to that of the planar [Tism^Pr^i^Benz^]^–^ anion with a comparable conformation (Fig. 3).

**Table 1:** Metrical data for [Tism^Pr^i^Benz^]M derivatives

	*d*(M–C)/Å	*d*(M–C) – ∑(cov. radii)^*a*^	∑(Si–C–Si)/°	*d*(C–[Si_3_])^*b*^/Å
[Tism^Pr^i^Benz^]Li	2.273(9)	0.23	355.8	0.22
[Tism^Pr^i^Benz^]MgMe	2.4925(12)	0.32	347.8	0.37
[κ^3^-Tism^Pr^i^Benz^]ZnMe	2.171(3)	0.19	346.3	0.40
[Tism^Pr^i^Benz^]Cu	2.281(7)	0.20	355.2	0.23
[κ^4^-*C*_4_-Tism^Pr^i^Benz*^]Cu	2.4283(18)	0.35	357.8	0.15
[Tism^Pr^i^Benz^]NiBr	2.2197(16)	0.22	347.7	0.38
[Tism^Pr^i^Benz^]H	—	—	342.3	0.47

^*a*^ Ref. 36.

^*b*^Distance of bridgehead carbon from the [Si_3_] plane.

**Scheme 2:**
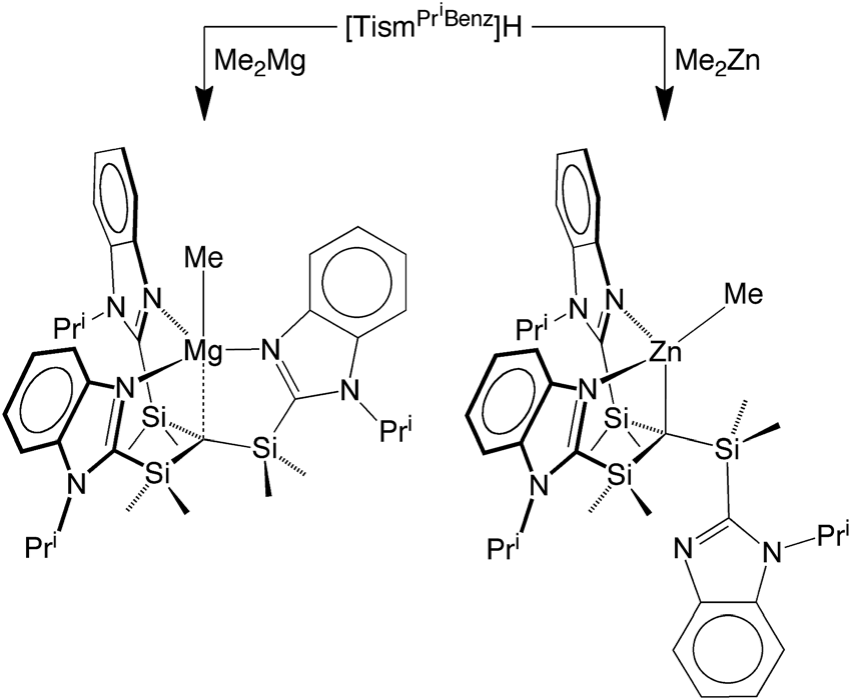


**Fig. 4:**
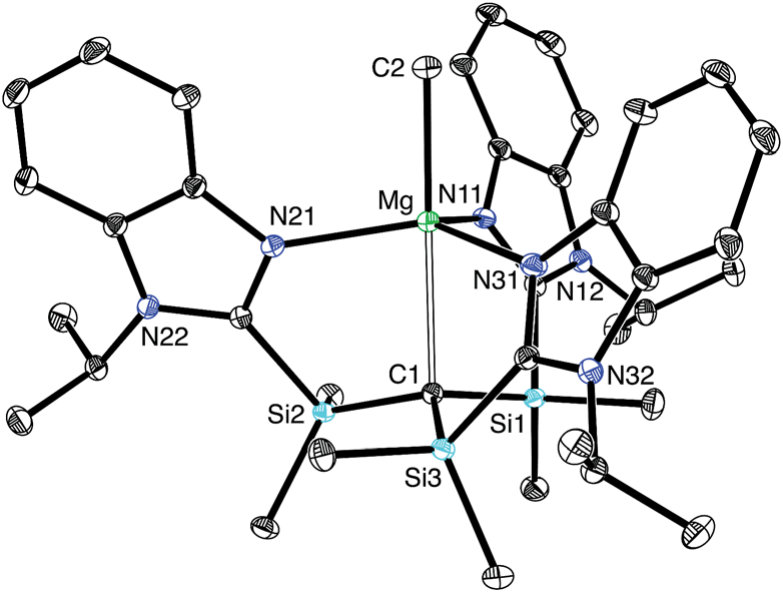
Molecular structure of [Tism^Pr^i^Benz^]MgMe.

**Fig. 5:**
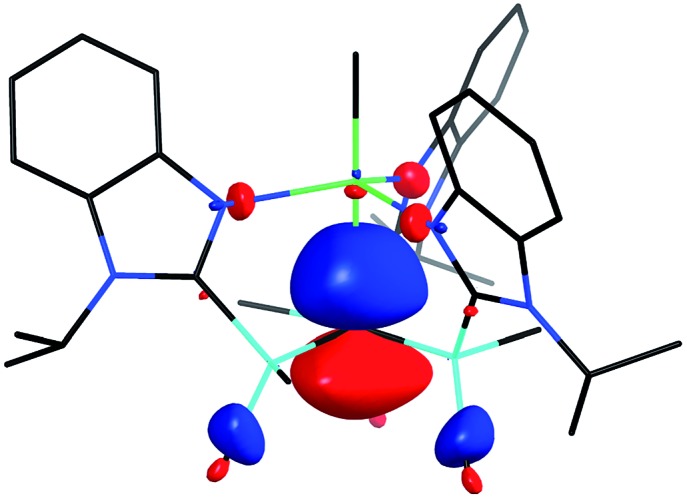
HOMO-1 of [Tism^Pr^i^Benz^]MgMe.

[Tism^Pr^i^Benz^]H and [Tism^Pr^i^Benz^]Li may be employed to form carbatrane compounds of the main group and transition metals. For example, [Tism^Pr^i^Benz^]H reacts with Me_2_Mg *via* elimination of methane to afford [Tism^Pr^i^Benz^]MgMe (Scheme 2). The molecular structure of [Tism^Pr^i^Benz^]MgMe has been determined by X-ray diffraction (Fig. 4), thereby demonstrating that the [Tism^Pr^i^Benz^] ligand coordinates in a κ^4^-manner such that the molecule possesses a carbatrane motif,^21^ but with a Mg–C_atrane_ distance [2.4925(12) Å] that is significantly longer (by 0.32 Å) than both (i) the Mg–CH_3_ bond length [2.1781(13) Å] and (ii) the sum of covalent radii (2.17 Å).^36^,^38^,^39^ The long Mg–C_atrane_ distance is, nevertheless, consistent with a zwitterionic description in which the carbon atom bears a formal negative charge. This qualitative view of the bonding is supported by computational studies which demonstrate that the HOMO-1 is effectively a lone pair orbital on carbon, with very little contribution from magnesium (Fig. 5).^40^ As such, the HOMO-1 of [Tism^Pr^i^Benz^]MgMe is similar in nature to the HOMO of [Tism^Pr^i^Benz^]Li. Despite the comparable atrane motifs of [Tism^Pr^i^Benz^]MgMe and [Tism^Pr^i^Benz^]Li, however, a notable difference is that the [CSi_3_] moiety of [Tism^Pr^i^Benz^]MgMe is more pyramidal than that of [Tism^Pr^i^Benz^]Li, as indicated by the fact that the sum of the Si–C–Si angles of [Tism^Pr^i^Benz^]MgMe (347.8°) is smaller than that for [Tism^Pr^i^Benz^]Li (355.8°).

**Fig. 6:**
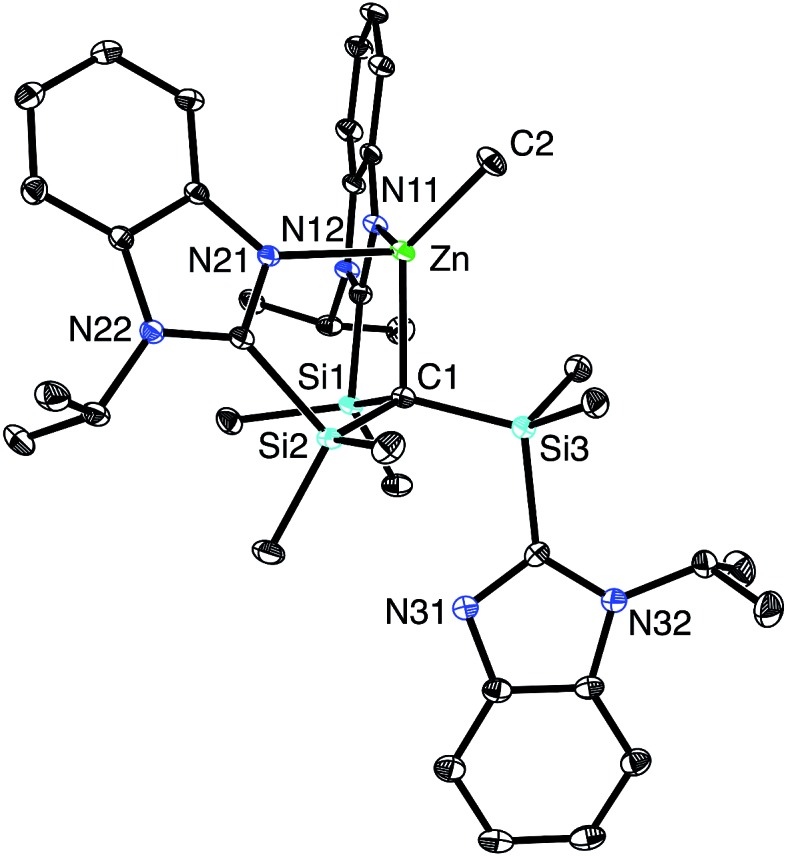
Molecular structure of [κ^3^-Tism^Pr^i^Benz^]ZnMe.

[Tism^Pr^i^Benz^]H can also be used as a reagent in zinc chemistry. Thus, [Tism^Pr^i^Benz^]H reacts with Me_2_Zn to afford [κ^3^-Tism^Pr^i^Benz^]ZnMe (Scheme 2). Although the reaction is analogous to that between [Tism^Pr^i^Benz^]H and Me_2_Mg, X-ray diffraction demonstrates that the zinc product, [κ^3^-Tism^Pr^i^Benz^]ZnMe, has a notably different structure to that of the magnesium counterpart. Specifically, rather than coordinating to zinc in a κ^4^-manner to afford a carbatrane motif, the ligand binds to zinc in a hypodentate^41^ κ^3^-manner, such that one of the imidazolyl groups remains uncoordinated (Fig. 6).^42^–^44^ Also in contrast to the magnesium derivative, [Tism^Pr^i^Benz^]MgMe, for which the Mg–CH_3_ and Mg–C_atrane_ bond lengths are very different, the corresponding bonds for [κ^3^-Tism^Pr^i^Benz^]ZnMe are more similar: *d*(Zn–CH_3_) = 1.989(3) Å and *d*(Zn–C_atrane_) = 2.171(3)Å, and the former is comparable to the sum of covalent radii (1.98 Å).^36^,^45^,^46^

**Fig. 7:**
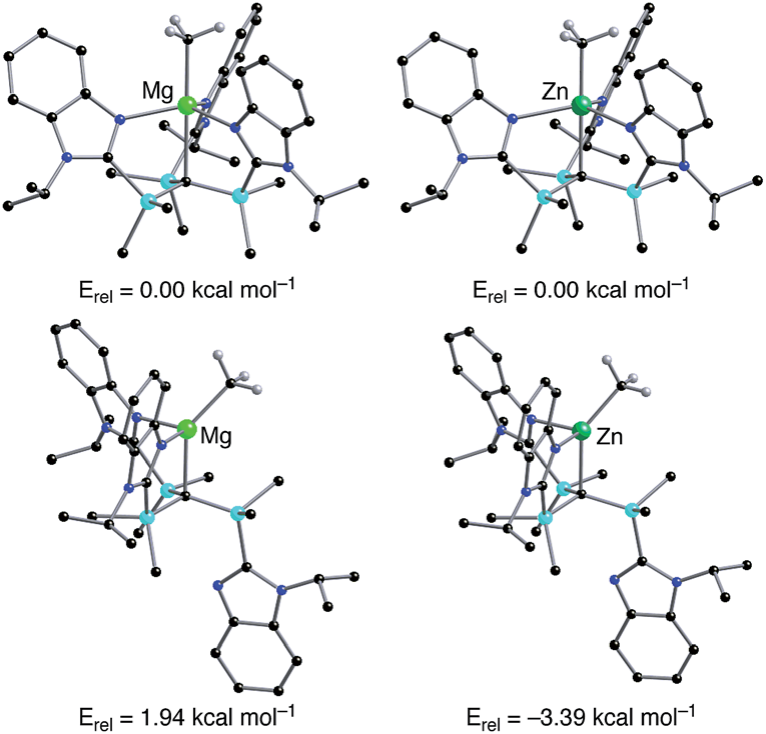
Relative energies of geometry optimized structures of κ^3^- and κ^4^-[Tism^Pr^i^Benz^]MMe (M = Mg, Zn). Hydrogen atoms on [Tism^Pr^i^Benz^] are omitted for clarity.

Density functional theory (DFT) calculations on the isomeric forms of [Tism^Pr^i^Benz^]MgMe and [Tism^Pr^i^Benz^]ZnMe support the experimental observations. Specifically, the DFT calculations demonstrate that the κ^4^-isomer is 1.94 kcal mol^–1^ more stable than the κ^3^-isomer for [Tism^Pr^i^Benz^]MgMe, whereas the κ^3^-isomer is 3.39 kcal mol^–1^ more stable than the κ^4^-isomer for [Tism^Pr^i^Benz^]ZnMe (Fig. 7).

**Scheme 3:**
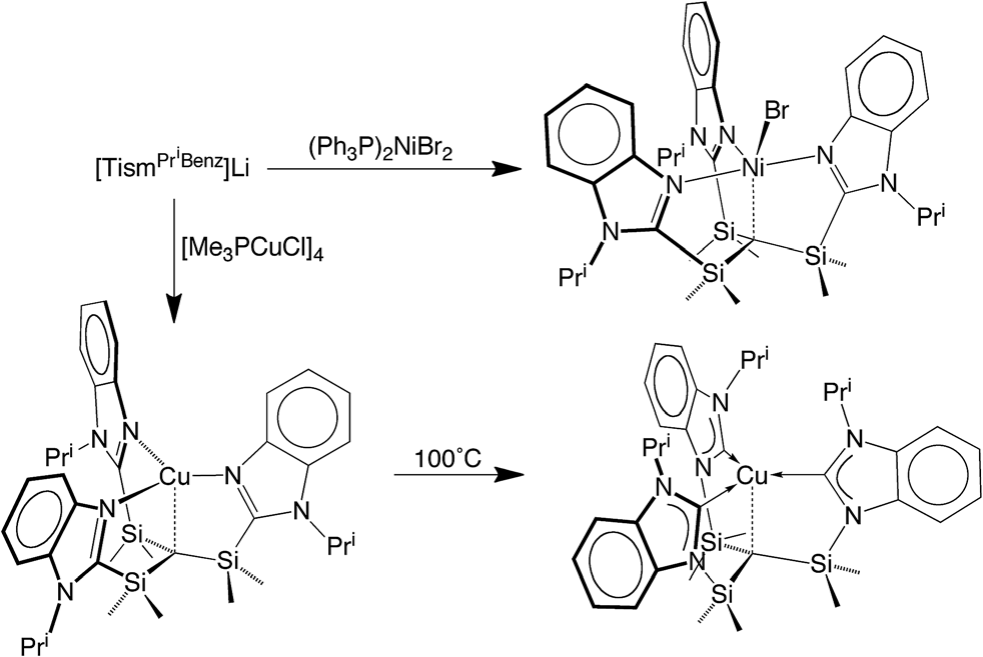


**Fig. 8:**
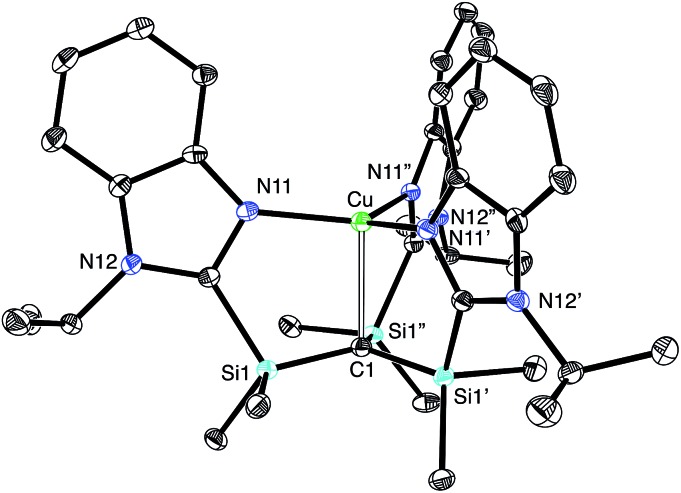
Molecular structure of [Tism^Pr^i^Benz^]Cu.

The lithium compound, [Tism^Pr^i^Benz^]Li, has also been used to synthesize metal complexes *via* metathesis reactions involving metal halides. For example, [Tism^Pr^i^Benz^]Li reacts with [(Me_3_P)CuCl]_4_ to give [Tism^Pr^i^Benz^]Cu (Scheme 3), which has been shown by X-ray diffraction (Fig. 8) to possess a trigonal monopyramidal structure similar to that of [Tism^Pr^i^Benz^]Li, with Cu–C and Cu–N distances of 2.281(7) Å and 2.014(3) Å, respectively.^47^,^48^ As with the lithium and magnesium carbatranes, the Cu–C_atrane_ bond of [Tism^Pr^i^Benz^]Cu is also longer (by 0.20 Å) than the sum of covalent radii (2.08 Å).^36^,^49^,^50^

**Fig. 9:**
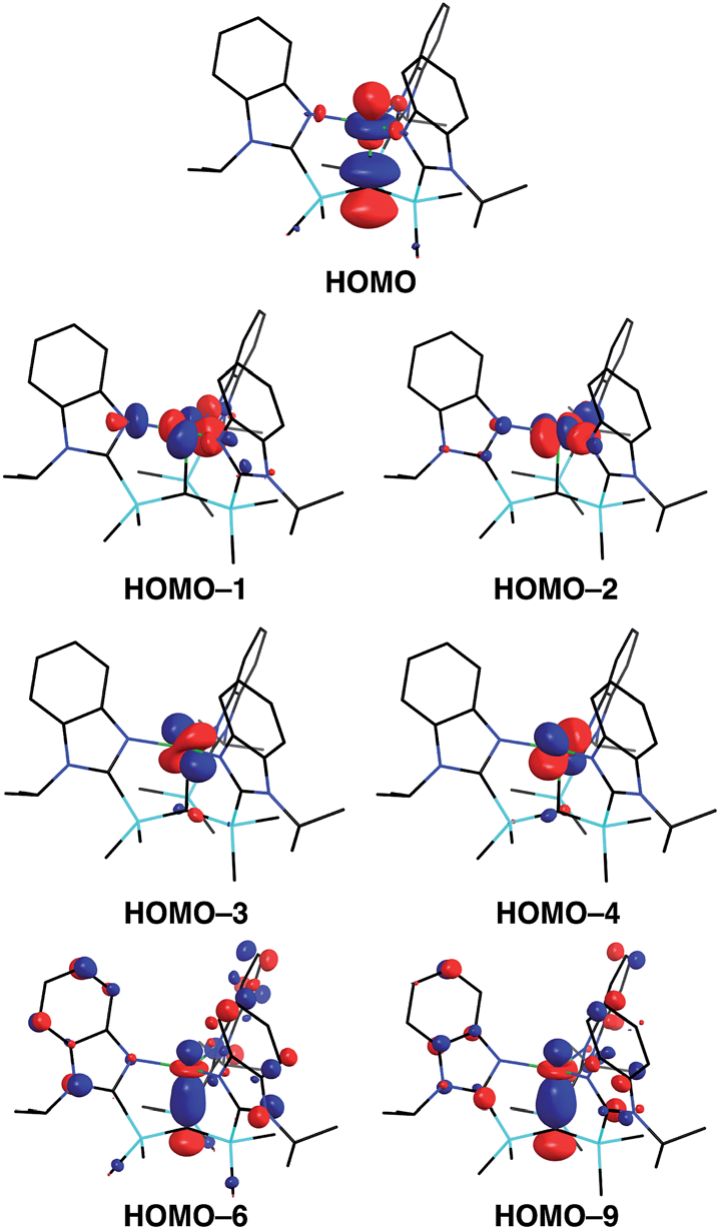
Frontier orbitals of [Tism^Pr^i^Benz^]Cu. Note that the in-phase interaction between the carbon 2p_z_ orbital and the copper 3d_z_^2^ orbital is a component of two molecular orbitals with similar energies (HOMO-6, –0.2169 eV; HOMO-9, –0.2192 eV).

**Fig. 10:**
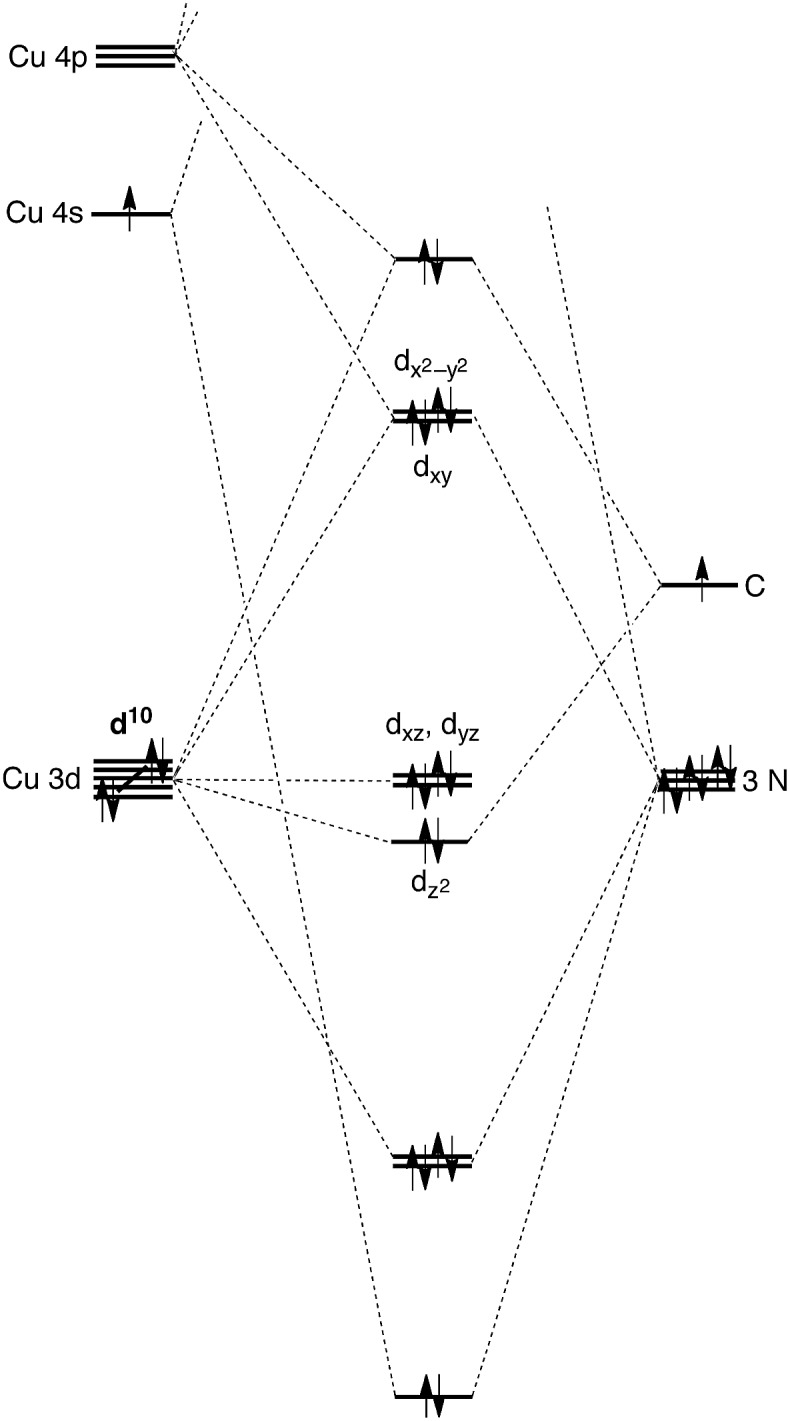
Qualitative molecular orbital diagram for [Tism^Pr^i^Benz^]Cu with *C*_3v_ symmetry, with the ligand arbitrarily represented in its neutral form.

A distinct difference between [Tism^Pr^i^Benz^]Cu and the lithium and magnesium complexes, [Tism^Pr^i^Benz^]Li and [Tism^Pr^i^Benz^]MgMe, however, is the degree of covalent interaction between copper and the atrane carbon atom. Specifically, overlap between the carbon 2p_*z*_ orbital and the copper 3d_*z*_^2^ orbital gives rise to Cu–C bonding and antibonding combinations, the latter of which is the HOMO (Fig. 9), as illustrated in the qualitative molecular orbital diagram shown in Fig. 10. Interestingly, the bonding combination possesses a significant copper component, while the antibonding combination possesses a significant carbon component; indeed, a natural bond orbital analysis of [Tism^Pr^i^Benz^]Cu classifies the HOMO as a carbon lone pair orbital. This arrangement is counter to that observed for most transition metal compounds with σ-donor ligands, for which the bonding combination usually possesses more ligand character because the ligand orbitals are typically lower in energy than the metal orbitals.^51^,^52^

However, despite the fact that situations in which the bonding orbital possesses mainly metal character (and the corresponding antibonding orbital possesses mainly ligand character) are not normally encountered in transition metal chemistry, examples of so-called “inverted ligand fields” have recently been discussed.^51^ Such circumstances may arise when the ligand σ-orbitals are higher in energy than the metal d orbitals, an occurrence that is more likely at the end of the transition series.^51^ A salient example is provided by [Cu(CF_3_)_4_]^–^, for which the bonding has been investigated both experimentally and computationally.^51^,^53^–^55^

**Fig. 11:**
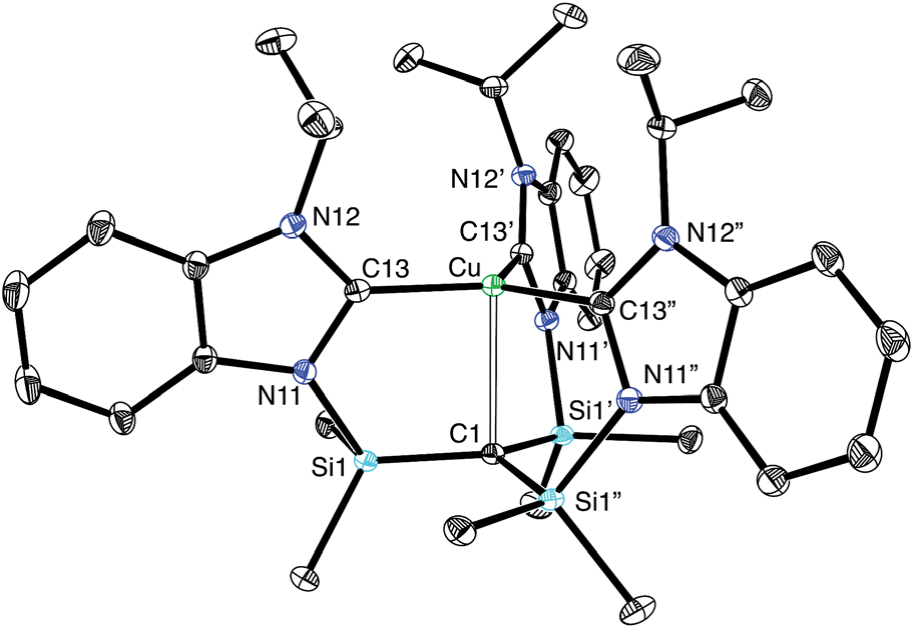
Molecular structure of [κ^4^-*C*_4_-Tism^Pr^i^Benz*^]Cu.

Most interestingly, [Tism^Pr^i^Benz^]Cu undergoes a novel isomerization at 100 °C to afford a tris(N-heterocyclic carbene) derivative, [κ^4^-*C*_4_-Tism^Pr^i^Benz*^]Cu (Scheme 3),^56^ which has been structurally characterized by X-ray diffraction (Fig. 11).

**Fig. 12:**
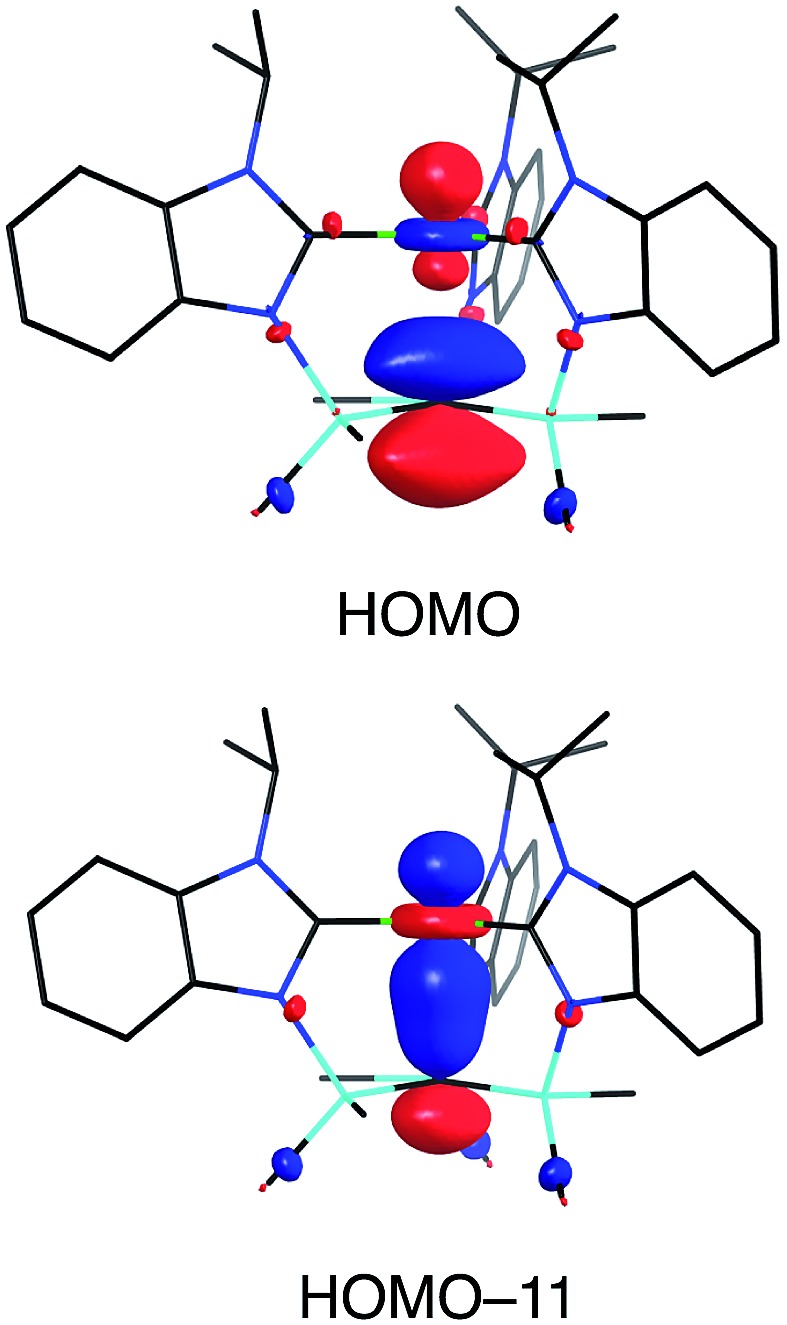
The HOMO and HOMO-11 of [κ^4^-*C*_4_-Tism^Pr^i^Benz*^]Cu.

Although several tripodal tris(N-heterocyclic carbene) ligands have been reported,^57^–^59^ the formation of [κ^4^-*C*_4_-Tism^Pr^i^Benz*^]Cu is notable because [κ^4^-*C*_4_-Tism^Pr^i^Benz*^] is an example of such a ligand that also features an additional potential X-type^7^ binding site.^60^ The isomerization of [Tism^Pr^i^Benz^]Cu to [κ^4^-*C*_4_-Tism^Pr^i^Benz*^]Cu is, however, accompanied by an increase in the axial Cu–C_atrane_ distance from 2.281(7) Å to 2.4283(18) Å, a value that is 0.35 Å longer than the sum of the covalent radii.^36^,^61^ Despite this lengthening, the copper 3d_z_^2^ and carbon 2p_z_ orbitals interact, and the derived bonding and antibonding orbitals are illustrated in Fig. 12. As observed for [Tism^Pr^i^Benz^]Cu, the antibonding orbital possesses mainly carbon character such that the bonding situation also corresponds to an “inverted ligand field”.

DFT calculations indicate that [κ^4^-*C*_4_-Tism^Pr^i^Benz*^]Cu is more stable than [Tism^Pr^i^Benz^]Cu by 4.18 kcal mol^–1^, which is in accord with the experimental observations. In contrast, isomerization of the structurally analogous lithium derivative, [Tism^Pr^i^Benz^]Li, to [κ^4^-*C*_4_-Tism^Pr^i^Benz*^]Li is predicted to be thermodynamically unfavorable by 35.3 kcal mol^–1^.^62^ The different thermodynamic trends reflect, *inter alia*, (i) the intrinsic stability of [Tism^Pr^i^Benz^] *versus* [Tism^Pr^i^Benz*^] and (ii) the relative preferences of copper and lithium to coordinate to a N-heterocyclic carbene *versus* an imidazole donor.^63^ With respect to the former, the tris(N-heterocyclic carbene), [Tism^Pr^i^Benz*^]H, is calculated to be 41.1 kcal mol^–1^ higher in energy than the tris(imidazole), [Tism^Pr^i^Benz^]H, with the 3 : 0 conformation^64^ that is used for κ^4^-coordination.^65^,^66^ As such, it is evident that coordination of the copper to the carbon donors provides a driving force for the isomerization.^67^ While the tautomerization of imidazoles to a C-coordinated ligand at a metal center has been previously observed,^68^–^70^ we are unaware of the corresponding transformation involving migration of a silyl group rather than a hydrogen atom.^71^ Furthermore, the formation of [κ^4^-*C*_4_-Tism^Pr^i^Benz*^]Cu is also noteworthy because C-coordination has been predicted to be less favorable than N-coordination of imidazole to CuCl.66a,b

**Fig. 13:**
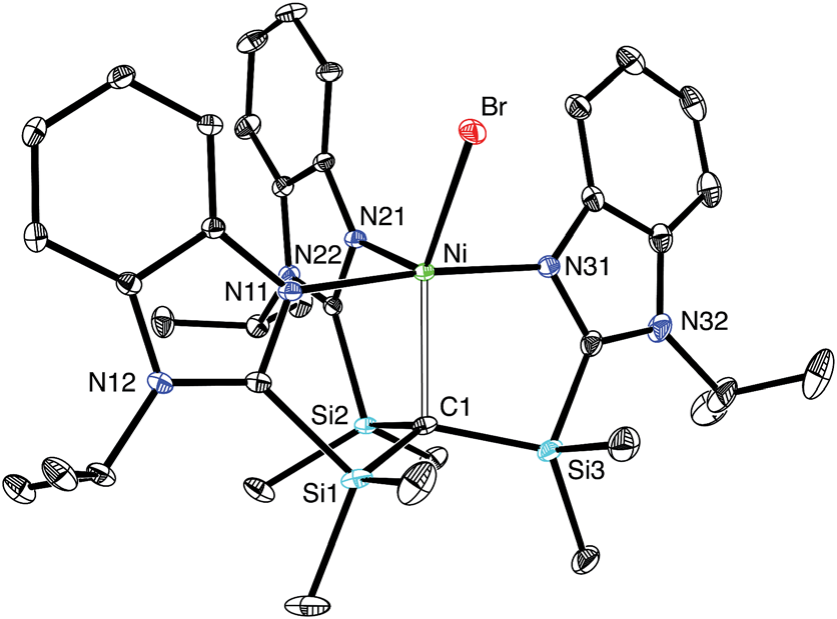
Molecular structure of [Tism^Pr^i^Benz^]NiBr.

The nickel compound, [Tism^Pr^i^Benz^]NiBr, may be obtained *via* metathesis of [Tism^Pr^i^Benz^]Li with (Ph_3_P)_2_NiBr_2_ (Scheme 3). Although the [Tism^Pr^i^Benz^] ligand binds in a κ^4^-manner (Fig. 13), with a Ni–C bond length of 2.2197(16) Å and Ni–N bond lengths in the range 2.0093(14)–2.1230(14) Å,^72^ the molecule does not adopt a trigonal bipyramidal structure akin to that of [Tism^Pr^i^Benz^]MgMe. Specifically, rather than possess three N–Ni–N angles of approximately 120°, the three nitrogen atoms of [Tism^Pr^i^Benz^]NiBr adopt a T-shaped arrangement, with N–Ni–N bond angles of 89.36(5)°, 90.34(6)° and 174.72(5)°; despite the different placement of the imidazole donors, however, the three C–Ni–N angles retain values of approximately 90°, namely 87.18(6)°, 87.63(6)° and 96.75(6)°.^72^ Thus, in addition to coordinating with a local *C*_3_ geometry, the [Tism^Pr^i^Benz^] ligand is flexible and may also coordinate with an idealized 90° seesaw geometry.^23^ Although the latter coordination mode of the [Tism^Pr^i^Benz^] ligand could support a square pyramidal structure, the location of the bromine is such that the coordination geometry of nickel is intermediate between square pyramidal and trigonal bipyramidal,^73^ as indicated by a *τ*_5_ five coordinate index of 0.44.^74^–^76^ As observed for the above carbatrane compounds, the Ni–C bond length [2.2197(16) Å] is also longer (by 0.22 Å) than the sum of covalent radii (2.00 Å).^36^,^77^

## Conclusions

In summary, [Tism^Pr^i^Benz^] is a flexible ligand that can coordinate to a metal center in both κ^3^ and κ^4^-manners, with the latter affording a carbatrane motif. Furthermore, when coordinating in a κ^4^-manner, the [Tism^Pr^i^Benz^] ligand can adopt either a trigonal monopyramidal geometry or a seesaw geometry. Interestingly, we have also demonstrated that the [Tism^Pr^i^Benz^] ligand may undergo a thermally induced rearrangement to afford a novel tripodal tris(N-heterocyclic carbene) ligand, as demonstrated by the conversion of [Tism^Pr^i^Benz^]Cu to [κ^4^-*C*_4_-Tism^Pr^i^Benz*^]Cu. A notable feature of the atrane compounds is that the transannular M–C bond lengths are 0.19–0.32 Å longer than the sum of the respective covalent radii, which is consistent with a zwitterionic component for the description of the molecules. Finally, a particularly noteworthy feature of both [Tism^Pr^i^Benz^]Cu and [κ^4^-*C*_4_-Tism^Pr^i^Benz*^]Cu is that Cu–C_atrane_ interaction is characterized by an “inverted ligand field”, in which the occupied antibonding orbital is more localized on carbon than on copper.

## Supplementary Material

Supplementary information

Crystal structure data
