# Supplementary material for: Tris[(1-isopropylbenzimidazol-2-yl)dimethylsilyl]methyl metal complexes, [TismPriBenz]M: a new class of metallacarbatranes, isomerization to a tris(N-heterocyclic carbene) derivative, and evidence for an inverted ligand field
Source: Chem Sci. 2017 May 2;8(6):4465–74. doi: 10.1039/c7sc00499k (PMC6100236; doi:10.1039/c7sc00499k)
Supplement: Supplementary file 1 [file SC-008-C7SC00499K-s001.pdf]

Electronic Supplementary Material (ESI) for Chemical Science.
This journal is © The Royal Society of Chemistry 2017

SUPPORTING INFORMATION

Tris[(1-isopropylbenzimidazol-2-yl)dimethylsilyl]methyl Metal Complexes, [Tism$^{Pr^iBenz}$]M: A New Class of Metallacarbatranes, Isomerization to a *Tris*(*N*-Heterocyclic Carbene) Derivative, and Evidence for an Inverted Ligand Field

Serge Ruccolo, Michael Rauch and Gerard Parkin,*

Department of Chemistry,

Columbia University,

New York, New York 10027, USA.

EXPERIMENTAL SECTION

General considerations

All manipulations were performed using a combination of glovebox, high vacuum, and Schlenk techniques under an argon atmosphere.[1] Solvents were purified and degassed by standard procedures. ^{1}H NMR chemical shifts are reported in ppm relative to $SiMe_4$ (δ = 0) and were referenced internally with respect to the protio solvent impurity (δ = 7.16 for C_6D_5H).[2] ^{13}C NMR spectra are reported in ppm relative to $SiMe_4$ (δ = 0) and were referenced internally with respect to the solvent (δ = 128.06 for C_6D_6).[2] ^{7}Li NMR are reported in ppm relative to LiCl (δ = 0) and were obtained by using the *Ξ*/100% value of 38.863797.[3] ^{29}Si NMR chemical shifts are reported in ppm relative to $SiMe_4$ (δ = 0.0) and were obtained by using the *Ξ*/100% value of 19.867187.[3] Coupling constants are given in hertz. Infrared spectra were recorded on a Perkin Elmer Spectrum Two spectrometer in attenuated total reflectance (ATR) mode and are reported in reciprocal centimeters. 1-isopropylbenzimidazole,[4] $HC(SiMe_2Cl)_3$,[5] Me_2Mg,[6] and $[Me_3PCuCl]_4$[7] were obtained by literature methods and Bu^nLi (Aldrich), MeLi (Aldrich), Me_2Zn (Aldrich), and $(Ph_3P)_2NiBr_2$ (Strem) were obtained commercially and used as received.

X-ray Structure Determinations

X-ray diffraction data were collected on a Bruker Apex II diffractometer. Crystal data, data collection and refinement parameters are summarized in Table 1. The structures were solved by using direct methods and standard difference map techniques, and were refined by full-matrix least-squares procedures on F^2 with SHELXTL (Version 2014/7).[8]

Computational Details

Calculations were carried out using DFT as implemented in the Jaguar 8.9 (release 15) suite of *ab initio* quantum chemistry programs.[9] Geometry optimizations were

performed with the B3LYP density functional[10] using the LACVP** basis sets. When required for thermodynamic comparisons, the energies of the optimized structures were re-evaluated by additional single point calculations on each optimized geometry using the cc-pVTZ(-f) correlation consistent triple (all atoms except Cu and Zn) and LACV3P** (Cu and Zn) basis sets.[11]

Synthesis of [Tism$^{Pr^iBenz}$]Li

The volatile components of a solution of MeLi in Et_2O (56.8 mmol, 35.5 mL of 1.6 M) were removed *in vacuo* and the solid obtained was dissolved in THF (*ca* 50 mL). The solution was treated slowly with distilled 1-isopropylbenzimidazole (8.81 g, 55 mmol) over a period of 10 minutes, and stirred for an additional 20 minutes. After this period, the volatile components were removed *in vacuo* to give an orange oil. Pentane (*ca* 30 mL) was added and the mixture was stirred for 10 minutes, after which period the volatile components were removed *in vacuo* to afford a light orange foam-like solid. The solid was dissolved in benzene (*ca* 30 mL) and the solution was added to a glass pressure vessel containing solid MeLi as obtained by the removal of volatile components *in vacuo* from a solution of MeLi in Et_2O (18.9 mmol, 11.8 mL of 1.6 M). The resulting orange suspension was placed in an ice bath and was treated slowly with a solution of freshly distilled $HC(SiMe_2Cl)_3$ (5.4 g, 18.4 mmol) in benzene (*ca* 20 mL) over a period of *ca.* 15 minutes. The mixture was allowed to warm to room temperature and stirred until gas evolution ceased (*ca* 1 hour), after which period the vessel was sealed and stirred at 100˚C for 15 hours (*CARE!*). Benzene (*ca* 40 mL) was added to the resulting red mixture, and the fine precipitate was allowed to settle and the solution was decanted. This process was repeated with two portions of benzene (*ca* 40 mL) and the combined extracts were concentrated *in vacuo* to a volume of *ca* 20 mL, thereby resulting in the deposition of a solid over a period of two days. The solid was isolated by filtration, washed with Et_2O (2 × *ca* 20 mL), and dried *in vacuo* to afford [Tism$^{Pr^iBenz}$]Li as an off-white powder, (5.1 g, 41 % yield). Colorless crystals of

$[Tism^{Pr^iBenz}]Li$ suitable for X-ray diffraction were obtained by slow evaporation of a solution in benzene. ^{1}H NMR (C_6D_6): 0.63 [s, 18H, $(C_6H_4N_2CH(CH_3)_2CSi(C\underline{H}_3)_2)_3CLi$], 1.23 [d, J = 7 Hz, 18H, $(C_6H_4N_2CH(C\underline{H}_3)_2CSi(CH_3)_2)_3CLi$], 4.84 [sept, J = 7 Hz, 3H, $(C_6H_4N_2C\underline{H}(CH_3)_2CSi(CH_3)_2)_3CLi$], 7.06 [m, 6H, $(C_6\underline{H}_4N_2CH(CH_3)_2CSi(CH_3)_2)_3CLi$], 7.28 [m, 3H, $(C_6\underline{H}_4N_2CH(CH_3)_2CSi(CH_3)_2)_3CLi$], 8.00 [m, 3H, $(C_6\underline{H}_4N_2CH(CH_3)_2CSi(CH_3)_2)_3CLi$]. ^{13}C NMR (C_6D_6): 6.23 [s, 6C, $(C_6H_4N_2CH(CH_3)_2CSi(\underline{C}H_3)_2)_3CLi$], 21.18 [s, 6C, $(C_6H_4N_2CH(\underline{C}H_3)_2CSi(CH_3)_2)_3CLi$], 49.40 [s, 3C, $(C_6H_4N_2\underline{C}H(CH_3)_2CSi(CH_3)_2)_3CLi$], 112.73 [s, 3C, $(\underline{C}_6H_4N_2CH(CH_3)_2CSi(CH_3)_2)_3CLi$], 119.78 [s, 3C, $(\underline{C}_6H_4N_2CH(CH_3)_2CSi(CH_3)_2)_3CLi$], 121.91 [s, 3C, $(\underline{C}_6H_4N_2CH(CH_3)_2CSi(CH_3)_2)_3CLi$], 121.96 [s, 3C, $(\underline{C}_6H_4N_2CH(CH_3)_2CSi(CH_3)_2)_3CLi$], 134.21 [s, 3C, $(\underline{C}_6H_4N_2CH(CH_3)_2CSi(CH_3)_2)_3CLi$], 145.52 [s, 3C, $(\underline{C}_6H_4N_2CH(CH_3)_2CSi(CH_3)_2)_3CLi$], 169.31 [s, 3C, $(C_6H_4N_2CH(CH_3)_2\underline{C}Si(CH_3)_2)_3CLi$], not observed [$(C_6H_4N_2CH(CH_3)_2CSi(CH_3)_2)_3\underline{C}Li$]. ^{29}Si NMR (C_6D_6): -14.48 [s, $(C_6H_4N_2CH(CH_3)_2C\underline{Si}(CH_3)_2)_3CLi$]. ^{7}Li NMR (C_6D_6): 5.32. Anal. calc. for $[Tism^{Pr^iBenz}]Li$: C, 66.2%; H, 7.7%; N, 12.5%. Found: C, 66.5%; H, 7.4%; N, 12.3%. IR Data (cm^{-1}): 3053 (w), 2964 (w), 2949 (w), 1463 (m), 1389 (w), 1371 (w), 1341 (m), 1320 (w), 1282 (m), 1159 (w), 1106 (w), 1061 (m), 964 (vs), 821 (s), 791 (s), 737 (vs), 672 (s).

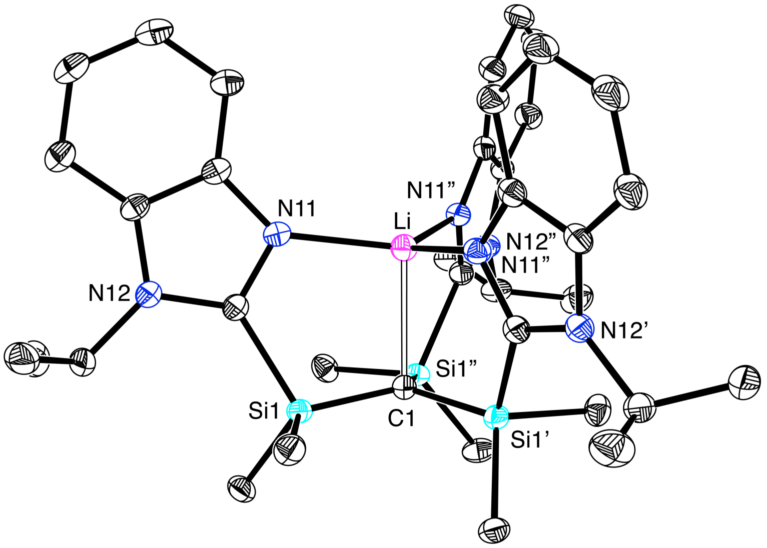

Molecular Structure $[Tism^{Pr^iBenz}]Li$

Synthesis of [Tism$^{Pr^iBenz}$]H

H_2O (40 μL, 2.22 mmol) was added to a solution of [Tism$^{Pr^iBenz}$]Li (640 mg, 0.954 mmol) in benzene (*ca* 25 mL). The mixture was stirred for 1 hour at room temperature, during which period a precipitate formed. The volatile components were removed *in vacuo* and the residue obtained was extracted into benzene (*ca* 25 mL). The solvent was removed from the extract *in vacuo* resulting in [Tism$^{Pr^iBenz}$]H as a white powder (610 mg, 96% yield). Colorless crystals of [Tism$^{Pr^iBenz}$]H suitable for X-ray diffraction were obtained *via* slow evaporation of a benzene solution. ^{1}H NMR (C_6D_6): 0.55 [s, 18H, $(C_6H_4N_2CH(CH_3)_2CSi(C\underline{H}_3)_2)_3CH$], 1.40 [d, J = 7 Hz, 18H, $(C_6H_4N_2CH(C\underline{H}_3)_2CSi(CH_3)_2)_3CH$], 2.30 [s, 1H, $(C_6H_4N_2CH(CH_3)_2CSi(CH_3)_2)_3C\underline{H}$], 5.09 [sep, J = 7 Hz, 3H, $(C_6H_4N_2C\underline{H}(CH_3)_2CSi(CH_3)_2)_3CH$], 7.14-7.23 [m, 6H, $(C_6\underline{H}_4N_2CH(CH_3)_2CSi(CH_3)_2)_3CH$], 7.38 [d, J = 8 Hz, 3H, $(C_6\underline{H}_4N_2CH(CH_3)_2CSi(CH_3)_2)_3CH$], 8.04 [d, J = 8 Hz, 3H, $(C_6\underline{H}_4N_2CH(CH_3)_2CSi(CH_3)_2)_3CH$]. ^{13}C{^{1}H} NMR ($C_6D_6$): -1.22 [s, 1C, $(C_6H_4N_2CH(CH_3)_2CSi(CH_3)_2)_3\underline{C}H$] , 1.89 [s, 6C, $(C_6H_4N_2CH(CH_3)_2CSi(\underline{C}H_3)_2)_3CH$], 21.49 [s, 6C, $(C_6H_4N_2CH(\underline{C}H_3)_2CSi(CH_3)_2)_3CH$], 49.85 [s, 3C, $(C_6H_4N_2\underline{C}H(CH_3)_2CSi(CH_3)_2)_3CH$], 112.69 [s, 3C, $(\underline{C}_6H_4N_2CH(CH_3)_2CSi(CH_3)_2)_3CH$], 121.13 [s, 3C, $(\underline{C}_6H_4N_2CH(CH_3)_2CSi(CH_3)_2)_3CH$], 121.73 [s, 3C, $(\underline{C}_6H_4N_2CH(CH_3)_2CSi(CH_3)_2)_3CH$], 122.49 [s, 3C, $(\underline{C}_6H_4N_2CH(CH_3)_2CSi(CH_3)_2)_3CH$], 134.84 [s, 3C, $(\underline{C}_6H_4N_2CH(CH_3)_2CSi(CH_3)_2)_3CH$], 146.98 [s, 3C, $(\underline{C}_6H_4N_2CH(CH_3)_2CSi(CH_3)_2)_3CH$], 159.75 [s, 3C, $(\underline{C}_6H_4N_2CH(CH_3)_2CSi(CH_3)_2)_3CH$]. ^{29}Si NMR ($C_6D_6$): -7.32 [s, 3Si, $(C_6H_4N_2CH(CH_3)_2C\underline{Si}(CH_3)_2)_3CH$]. IR Data (cm^{-1}): 2979 (w), 1459 (m), 1394 (w), 1340 (s), 1321 (m), 1266 (m) 1162 (w), 1131 (w) 1105 (w), 1060 (m), 1014 (m), 834 (s), 820 (vs), 775 (s), 740 (vs), 681 (vs).

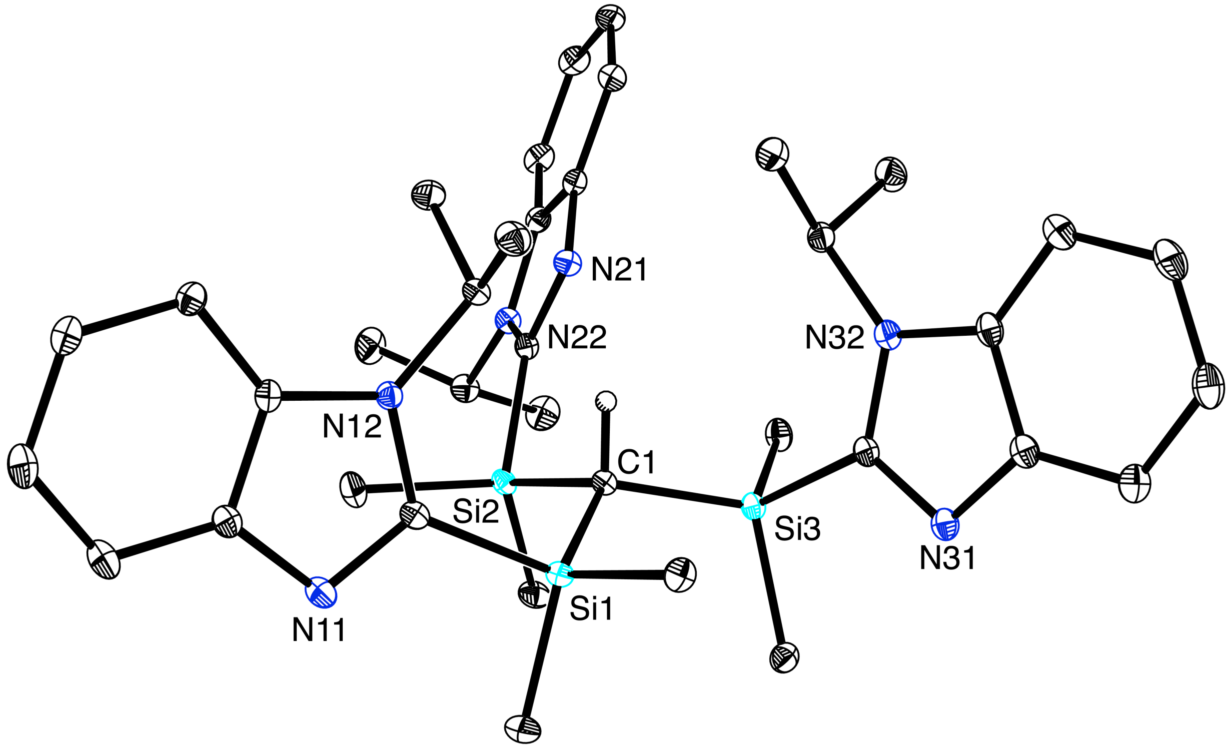

Molecular Structure of $[\mathrm{Tism}^{\mathrm{Pr^iBenz}}]\mathrm{H}\bullet 1.5(C_6H_6)$ *(solvent not shown for clarity)*

Conversion of $[\mathrm{Tism}^{\mathrm{Pr^iBenz}}]\mathrm{H}$ to $[\mathrm{Tism}^{\mathrm{Pr^iBenz}}]\mathrm{Li}$

A solution of $[\mathrm{Tism}^{\mathrm{Pr^iBenz}}]\mathrm{H}$ (30 mg, 0.045 mmol) in THF (*ca* 2 mL) was treated with $\mathrm{Bu^nLi}$ (0.05 mL, 1.6 M in hexanes). The mixture was stirred for one hour, during which period the solution turned light orange. The volatile components were removed *in vacuo* and the formation of $[\mathrm{Tism}^{\mathrm{Pr^iBenz}}]\mathrm{Li}$ was demonstrated by ^{1}H NMR spectroscopy.

Synthesis of $[\mathrm{Tism}^{\mathrm{Pr^iBenz}}]\mathrm{MgMe}$

A solution of $[\mathrm{Tism}^{\mathrm{Pr^iBenz}}]\mathrm{H}$ (400 mg, 0.601 mmol) in benzene (*ca* 20 mL) was treated with Me_2Mg (40 mg, 0.736 mmol). The mixture was stirred for 20 minutes, after which the mixture was filtered and the filtrate was lyophilized to afford $[\mathrm{Tism}^{\mathrm{Pr^iBenz}}]\mathrm{MgMe}$ as a white powder (405 mg, 96% yield). Colorless crystals of $[\mathrm{Tism}^{\mathrm{Pr^iBenz}}]\mathrm{MgMe}$ suitable for X-ray diffraction were obtained *via* slow evaporation of a benzene solution. ^{1}H NMR (C_6D_6): 0.20 [s, 3H, $(C_6H_4N_2CH(CH_3)_2CSi(CH_3)_2)_3CMgC\underline{H}_3$], 0.45 [s, 18H, $(C_6H_4N_2CH(CH_3)_2CSi(C\underline{H}_3)_2)_3CMgCH_3$], 1.16 [d, J = 7 Hz, 18H,

$(C_6H_4N_2CH(C\underline{H}_3)_2CSi(CH_3)_2)_3CMgCH_3]$, 4.66 [sep, J = 7 Hz, 3H, $(C_6H_4N_2C\underline{H}(CH_3)_2CSi(CH_3)_2)_3CMgCH_3]$, 7.01-7.19 [m, 9H, $(C_6\underline{H}_4N_2CH(CH_3)_2CSi(CH_3)_2)_3CMgCH_3]$, 8.83 [d, J = 8 Hz, 3H, $(C_6\underline{H}_4N_2CH(CH_3)_2CSi(CH_3)_2)_3CMgCH_3]$. $^{13}C\{^1H\}$ NMR (C_6D_6): 0.57 [s, 1C, $(C_6H_4N_2CH(CH_3)_2CSi(CH_3)_2)_3CMg\underline{C}H_3]$, 4.54 [s, 6C, $(C_6H_4N_2CH(CH_3)_2CSi(\underline{C}H_3)_2)_3CMgCH_3]$, 21.01 [s, 6C, $(C_6H_4N_2CH(\underline{C}H_3)_2CSi(CH_3)_2)_3CMgCH_3]$, 50.09 [s, 3C, $(C_6H_4N_2\underline{C}H(CH_3)_2CSi(CH_3)_2)_3CMgCH_3]$, 112.24 [s, 3C, $(\underline{C}_6H_4N_2CH(CH_3)_2CSi(CH_3)_2)_3CMgCH_3]$, 122.54 [s, 3C, $(\underline{C}_6H_4N_2CH(CH_3)_2CSi(CH_3)_2)_3CMgCH_3]$, 122.69 [s, 3C, $(\underline{C}_6H_4N_2CH(CH_3)_2CSi(CH_3)_2)_3CMgCH_3]$, 122.90 [s, 3C, $(\underline{C}_6H_4N_2CH(CH_3)_2CSi(CH_3)_2)_3CMgCH_3]$, 134.55 [s, 3C, $(\underline{C}_6H_4N_2CH(CH_3)_2CSi(CH_3)_2)_3CMgCH_3]$, 144.78 [s, 3C, $(\underline{C}_6H_4N_2CH(CH_3)_2CSi(CH_3)_2)_3CMgCH_3]$, 166.94 [s, 3C, $(\underline{C}_6H_4N_2CH(CH_3)_2CSi(CH_3)_2)_3CMgCH_3]$, not observed $[(C_6H_4N_2CH(CH_3)_2CSi(CH_3)_2)_3\underline{C}MgCH_3]$. ^{29}Si NMR (C_6D_6): -12.69 [s, 3Si, $(C_6H_4N_2CH(CH_3)_2C\underline{Si}(CH_3)_2)_3CMgCH_3]$. Anal. calc. for $[Tism^{Pr^iBenz}]MgMe\bullet C_6H_6$: C, 67.6%; H, 7.7%; N, 10.8%. Found: C, 66.8%; H, 7.6%; N, 10.4%. IR Data (ATR, cm^{-1}): 2973 (m), 1462 (m), 1391 (w), 1340 (m), 1257 (m), 1060 (m), 1011 (m), 948 (s), 821 (vs), 739 (vs), 676 (s).

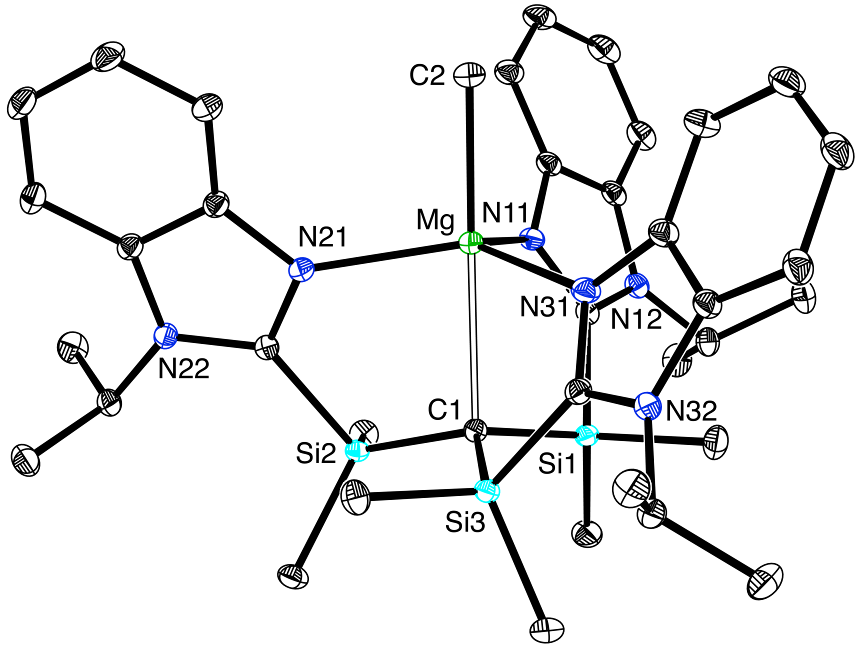

Molecular Structure of [Tism$^{Pr^iBenz}$]MgMe•(C_6H_6) (solvent not shown for clarity)

Synthesis of [κ^3-Tism$^{Pr^iBenz}$]ZnMe

A solution of [Tism$^{Pr^iBenz}$]H (30 mg, 0.045 mmol) in benzene (*ca.* 0.7 mL) was treated with Me_2Zn (15 mg, 0.157 mmol) in an NMR tube equipped with a J. Young valve and the solution was heated at 60°C for 1 day. After this period the mixture was filtered and the filtrate was lyophilized to afford [κ^3-Tism$^{Pr^iBenz}$]ZnMe as a white powder (18 mg, 54%). Colorless crystals of [κ^3-Tism$^{Pr^iBenz}$]ZnMe suitable for X-ray diffraction were obtained by vapor diffusion of pentane into a solution in benzene. ^{1}H NMR (C_6D_6): 0.31 [s, 3H, $(C_6H_4N_2CH(CH_3)_2CSi(CH_3)_2)_3CZnC\underline{H}_3$], 0.76 [s, 18H, $(C_6H_4N_2CH(CH_3)_2CSi(C\underline{H}_3)_2)_3CZnCH_3$], 1.14 [d, J = 6.9 Hz, 18H, $(C_6H_4N_2CH(C\underline{H}_3)_2CSi(CH_3)_2)_3CZnCH_3$], 4.70 [sept, J = 7 Hz, 3H $(C_6H_4N_2C\underline{H}(CH_3)_2CSi(CH_3)_2)_3CZnCH_3$], 7.05 [m, 3H, $(C_6\underline{H}_4N_2CH(CH_3)_2CSi(CH_3)_2)_3CZnCH_3$], 7.23 [d, J = 8.2 Hz, $(C_6\underline{H}_4N_2CH(CH_3)_2CSi(CH_3)_2)_3CZnCH_3$], 8.22 [d, J = 8 Hz, $(C_6\underline{H}_4N_2CH(CH_3)_2CSi(CH_3)_2)_3CZnCH_3$]. ^{13}C NMR ($C_6D_6$): -10.51 [s, 1C, $(C_6H_4N_2CH(CH_3)_2CSi(CH_3)_2)_3CZn\underline{C}H_3$], 4.98 [s, 6C,

$(C_6H_4N_2CH(CH_3)_2CSi(\underline{C}H_3)_2)_3CZnCH_3]$, 21.15 [s, 6C, $(C_6H_4N_2CH(\underline{C}H_3)_2CSi(CH_3)_2)_3CZnCH_3]$, 49.97 [s, 3C, $(C_6H_4N_2\underline{C}H(CH_3)_2CSi(CH_3)_2)_3CZnCH_3]$, 112.92 [s, 3C, $(\underline{C}_6H_4N_2CH(CH_3)_2CSi(CH_3)_2)_3CZnCH_3]$, 119.85 [s, 3C, $(\underline{C}_6H_4N_2CH(CH_3)_2CSi(CH_3)_2)_3CZnCH_3]$, 122.38 [s, 3C, $(\underline{C}_6H_4N_2CH(CH_3)_2CSi(CH_3)_2)_3CZnCH_3]$, 122.50 [s, 3C, $(\underline{C}_6H_4N_2CH(CH_3)_2CSi(CH_3)_2)_3CZnCH_3]$, 134.68 [s, 3C, $(\underline{C}_6H_4N_2CH(CH_3)_2CSi(CH_3)_2)_3CZnCH_3]$, 144.59 [s, 3C, $(\underline{C}_6H_4N_2CH(CH_3)_2CSi(CH_3)_2)_3CZnCH_3]$, 166.41 [s, 3C, $(C_6H_4N_2CH(CH_3)_2\underline{C}Si(CH_3)_2)_3CZnCH_3]$, not observed $[(C_6H_4N_2CH(CH_3)_2CSi(CH_3)_2)_3\underline{C}ZnCH_3]$. ^{29}Si NMR (C_6D_6): -7.97 [s, $(C_6H_4N_2CH(CH_3)_2C\underline{Si}(CH_3)_2)_3CZnCH_3]$. Anal. calc. for $[\kappa^3\text{-Tism}^{Pr^iBenz}]ZnMe$: C, 61.3%; H, 7.3%; N, 11.3%. Found: C, 61.7%; H, 6.7%; N, 10.2%. IR Data (cm^{-1}): 2969 (w), 2896 (w), 1464 (m), 1390 (m), 1371 (m), 1354 (m), 1290 (w), 1252 (m), 1132 (w), 1066 (m), 919 (s), 890 (s), 813 (s), 739 (vs), 698 (m), 645 (m).

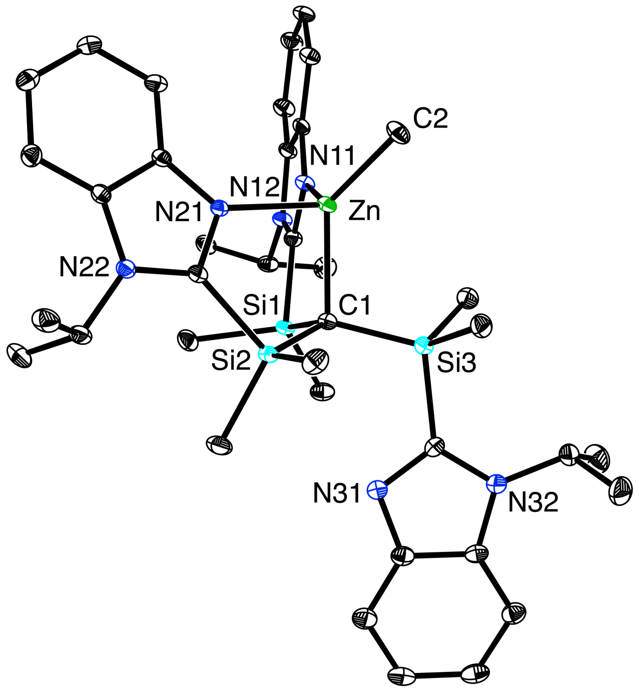

Molecular Structure of $[k^3\text{-Tism}^{Pr^iBenz}]ZnMe \bullet (C_6H_6)$ (solvent not shown for clarity)

Synthesis of $[Tism^{Pr^iBenz}]Cu$

A solution of $[(Me_3P)CuCl]_4$ (16 mg, 0.023 mmol) in benzene (*ca.* 1.5 mL) was treated with $[Tism^{Pr^iBenz}]Li$ (50 mg, 0.075 mmol), thereby resulting in the formation of a yellow suspension. The suspension was stirred for one day, after which period, the solvent was removed *in vacuo*. The solid was washed with benzene (2 × 0.5 mL), resulting in $[Tism^{Pr^iBenz}]Cu$ as a yellow solid (16 mg, 30 %). Yellow crystals suitable for X-ray diffraction were obtained by vapor diffusion of pentane into a solution in benzene. ^1H NMR (C_6D_6) 0.58 [s, 18H, $(C_6H_4N_2CH(CH_3)_2CSi(C\underline{H}_3)_2)_3CCu$], 1.23 [d, J = 7 Hz, 18H, $(C_6H_4N_2CH(C\underline{H}_3)_2CSi(CH_3)_2)_3CCu$], 4.77 [sept, J = 7 Hz, 3H, $(C_6H_4N_2C\underline{H}(CH_3)_2CSi(CH_3)_2)_3CCu$], 7.02 [m, 3H, $(C_6\underline{H}_4N_2CH(CH_3)_2CSi(CH_3)_2)_3CCu$], 7.11 [m, 3H, $(C_6\underline{H}_4N_2CH(CH_3)_2CSi(CH_3)_2)_3CCu$], 7.26 [d, J = 8.1 Hz, 3H, $(C_6\underline{H}_4N_2CH(CH_3)_2CSi(CH_3)_2)_3CCu$], 8.29 [d, J = 8 Hz, 3H, $(C_6\underline{H}_4N_2CH(CH_3)_2CSi(CH_3)_2)_3CCu$]. ^{13}C NMR (C_6D_6): 5.65 (6C, $(C_6H_4N_2CH(CH_3)_2CSi(\underline{C}H_3)_2)_3CCu$], 21.24 [6C, $(C_6H_4N_2CH(\underline{C}H_3)_2CSi(CH_3)_2)_3CCu$], 49.64 [3C, $(C_6H_4N_2\underline{C}H(CH_3)_2CSi(CH_3)_2)_3CCu$], 112.64 [3C, $(\underline{C}_6H_4N_2CH(CH_3)_2CSi(CH_3)_2)_3CCu$], 120.21 [3C, $(\underline{C}_6H_4N_2CH(CH_3)_2CSi(CH_3)_2)_3CCu$], 121.91 [3C, $(\underline{C}_6H_4N_2CH(CH_3)_2CSi(CH_3)_2)_3CCu$], 122.02 [3C, $(\underline{C}_6H_4N_2CH(CH_3)_2CSi(CH_3)_2)_3CCu$], 134.13 [3C, $(\underline{C}_6H_4N_2CH(CH_3)_2CSi(CH_3)_2)_3CCu$], 144.34 [3C, $(\underline{C}_6H_4N_2CH(CH_3)_2CSi(CH_3)_2)_3CCu$], 168.12 [3C, $(\underline{C}_6H_4N_2CH(CH_3)_2CSi(CH_3)_2)_3CCu$], not observed [$(C_6H_4N_2CH(CH_3)_2CSi(CH_3)_2)_3\underline{C}Cu$]. ^{29}Si NMR (C_6D_6): -14.90 [s, 3Si, $(C_6H_4N_2CH(CH_3)_2C\underline{Si}(CH_3)_2)_3CCu$] . Anal. calc. for $[Tism^{Pr^iBenz}]Cu$: C, 61.1%; H, 7.1%; N, 11.6%. Found: C, 60.8%; H, 7.0%; N, 11.4%. IR Data (cm^{-1}): 3053 (w), 2963 (w), 1463 (m), 1389 (w), 1372 (w), 1344 (m), 1321 (w), 1286 (w), 1249 (m), 1159 (w), 1133 (w), 1105 (m), 954 (vs), 822 (s), 791 (s), 738 (vs), 669 (m).

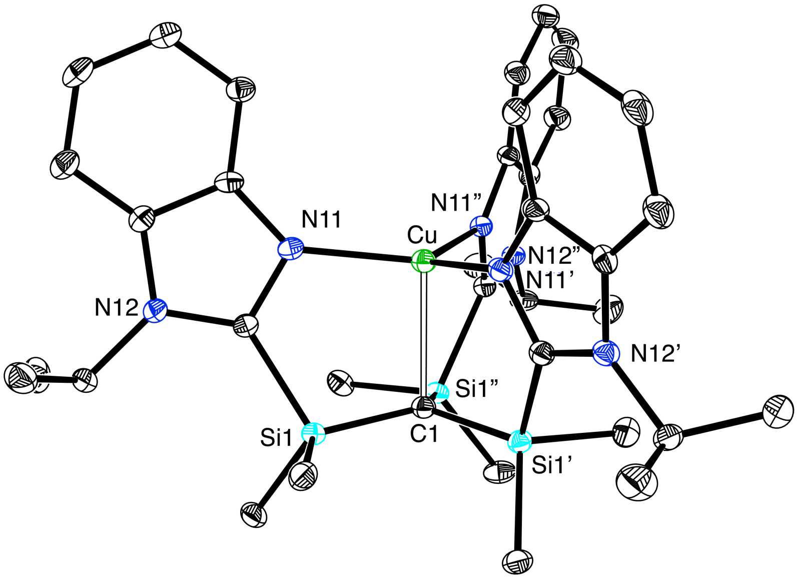

Molecular Structure of [Tism$^{Pr^iBenz}$]Cu

Synthesis of [κ^4-*C*$_4$-Tism$^{Pr^iBenz*}$]Cu

(i) A solution of [Tism$^{Pr^iBenz}$]Li (20 mg, 0.03 mmol) in benzene (*ca.* 0.4 mL) in an NMR tube equipped with a J. Young valve was treated with [$(Me_3P)CuCl]_4$ (8.5 mg, 0.012 mmol). The solution was heated at 100°C for 3 hours. Upon cooling, large crystals of [κ^4-*C*$_4$-Tism$^{Pr^iBenz*}$]Cu suitable for X-ray diffraction were deposited and isolated. Slow evaporation of the mother liquor deposited additional crystals of [κ^4-*C*$_4$-Tism$^{Pr^iBenz*}$]Cu which were collected, washed with pentane and dried *in vacuo* (16 mg, 73%). ^{1}H NMR (C_6D_6): 0.61 [s, 18H, $(C_6H_4CNCH(CH_3)_2NSi(C\underline{H}_3)_2)_3CCu$], 1.46 [d, J = 7 Hz, 18H, $(C_6H_4CNCH(C\underline{H}_3)_2NSi(CH_3)_2)_3CCu$], 5.34 [sept, J = 7 Hz, 3H, $(C_6H_4CNC\underline{H}(CH_3)_2NSi(CH_3)_2)_3CCu$], 7.01 [m, 6H, $(C_6\underline{H}_4CNCH(CH_3)_2NSi(CH_3)_2)_3CCu$], 7.20 [m, 3H, $(C_6\underline{H}_4CNCH(CH_3)_2NSi(CH_3)_2)_3CCu$], 7.56 [m, 3H, $(C_6\underline{H}_4CNCH(CH_3)_2NSi(CH_3)_2)_3CCu$]. ^{13}C NMR ($C_6D_6$): 5.72 [s, 6C, $(C_6H_4CNCH(CH_3)_2NSi(\underline{C}H_3)_2)_3CCu$], 21.56 [s,6C, 7.20 [m, 6H, $(C_6H_4CNCH(\underline{C}H_3)_2NSi(CH_3)_2)_3CCu$], 53.31 [s, 3C, $(C_6H_4CN\underline{C}H(CH_3)_2NSi(CH_3)_2)_3CCu$], 111.08 [s, 3C, $(\underline{C}_6H_4CNCH(CH_3)_2NSi(CH_3)_2)_3CCu$], 113.57 [s, 3C, $(\underline{C}_6H_4CNCH(CH_3)_2NSi(CH_3)_2)_3CCu$], 120.80 [s, 3C, $(\underline{C}_6H_4CNCH(CH_3)_2NSi(CH_3)_2)_3CCu$],

121.85 [s, 3C, ($\underline{C}_6H_4CNCH(CH_3)_2NSi(CH_3)_2)_3CCu$], 134.95 [s, 3C, ($\underline{C}_6H_4CNCH(CH_3)_2NSi(CH_3)_2)_3CCu$], 139.50 [s, 3C, ($\underline{C}_6H_4CNCH(CH_3)_2NSi(CH_3)_2)_3CCu$], 207.73 [s, 3C, ($C_6H_4\underline{C}NCH(CH_3)_2NSi(CH_3)_2)_3CCu$], not observed [$(C_6H_4CNCH(CH_3)_2NSi(CH_3)_2)_3\underline{C}Cu$]. ^{29}Si NMR ($C_6D_6$): 4.57 [s, ($C_6H_4CNCH(CH_3)_2N\underline{Si}(CH_3)_2)_3CCu$]. Anal. calc. for [$\kappa^4$-$C_4$-Tism$^{Pr^iBenz*}$]Cu•0.33($C_6H_6$): C, 63.2%; H, 7.1%; N, 10.8%. Found: C, 62.2%; H, 6.9%; N, 10.7%. IR Data (cm^{-1}): 2973 (w), 1470 (m), 1388 (w), 1350 (m), 1282 (vs), 1251 (m), 1219 (m), 1166 (w), 1067 (m), 970 (vs), 811 (m), 793 (m), 768 (m), 741 (vs), 681 (m).

(ii) A solution of [Tism$^{Pr^iBenz}$]Cu (2 mg) in C_6D_6 (*ca.* 0.5 mL) in an NMR tube equipped with a J. Young valve, containing mesitylene as an internal standard, was heated at 100˚C for 30 minutes. The sample was monitored by ^{1}H NMR spectroscopy, thereby demonstrating conversion to [κ^4-C_4-Tism$^{Pr^iBenz*}$]Cu in 80% yield.

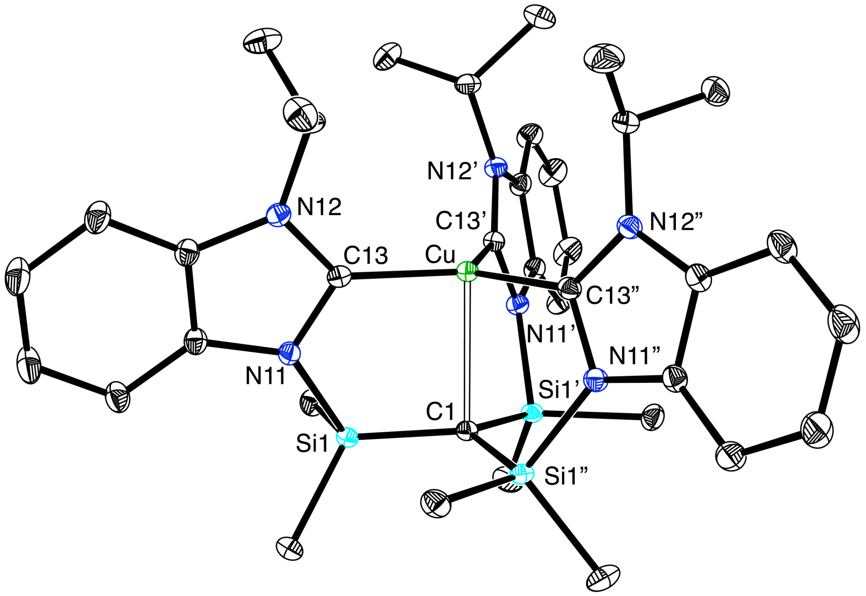

Molecular Structure of [κ^4-C_4-Tism$^{Pr^iBenz*}$]Cu•0.5(C_6H_6) (solvent not shown for clarity)

Synthesis of [Tism$^{Pr^iBenz}$]NiBr

A mixture of [Tism$^{Pr^iBenz}$]Li (50 mg, 0.075 mmol) and $(Ph_3P)_2NiBr_2$ (55 mg, 0.074 mmol) was dissolved in THF (*ca.* 2 mL) and the green solution obtained was allowed to stand over a period of 2 days, during which period green crystals of [Tism$^{Pr^iBenz}$]NiBr suitable for X-ray diffraction were deposited. The crystals were isolated by decanting the mother liquor, washed sequentially with THF (*ca.* 1 mL) and pentane (*ca.* 1 mL), and dried *in vacuo* to afford [Tism$^{Pr^iBenz}$]NiBr (37 mg, 61%). ^{1}H NMR (C_6D_6): 1.53 [bs], 4.85 [bs], 5.59 [bs], 8.09 [bs], 10.21 [bs], 22.52 [bs], 29.88 [bs]. Anal. calc. for [Tism$^{Pr^iBenz}$]NiBr: C, 55.4%; H, 6.4%; N, 10.5%. Found: C, 54.1%; H, 6.6%; N, 9.1%. IR Data (cm^{-1}): 2972 (w), 2943 (w), 2877 (w), 1466 (m) 1392 (m), 1363 (m), 1252 (m), 1158 (w), 1133 (w), 1068 (m), 933 (s), 896 (s), 829 (s), 815 (s), 766 (m), 737 (vs), 695 (m).

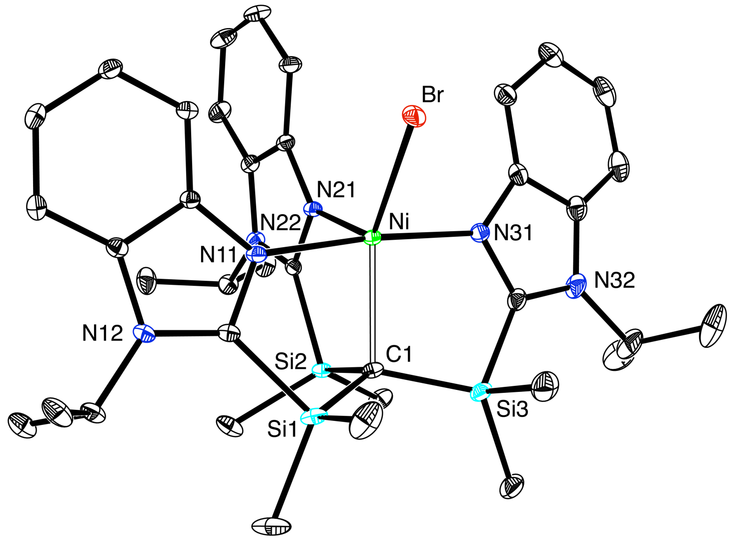

Molecular structure of [Tism$^{Pr^iBenz}$]NiBr•THF (solvent not shown for clarity)

Table 1. Crystal, intensity collection, and refinement data.

	**[Tism$^{Pr^iBenz}$]Li**	**[Tism$^{Pr^iBenz}$]H•1.5(C_6H_6)**
lattice	Cubic	Triclinic
formula	$C_{37}H_{51}LiN_6Si_3$	$C_{46}H_{61}N_6Si_3$
formula weight	671.04	782.27
space group	*I23*	*P-1*
a/Å	20.191(2)	10.1605(10)
b/Å	20.191(2)	15.4631(16)
c/Å	20.191(2)	15.8502(16)
α/°	90	65.3205(14)
β/°	90	86.4167(16)
γ/°	90	79.4613(15)
V/Å^3	8231(3)	2224.4(4)
Z	8	2
temperature (K)	130(2)	130(2)
radiation (λ, Å)	0.71073	0.71073
ρ (calcd.) g cm^{-3}	1.083	1.168
μ (Mo Kα), mm^{-1}	0.146	0.145
θ max, deg.	30.509	30.625
no. of data collected	65610	36500
no. of data	4198	13593
no. of parameters	146	512
R_1 [$I > 2\sigma(I)$]	0.0438	0.0550
wR_2 [$I > 2\sigma(I)$]	0.1164	0.1339
R_1 [all data]	0.0558	0.0689
wR_2 [all data]	0.1285	0.1402
GOF	0.953	1.090
R_{int}	0.0576	0.0298

Table 1. (cont.) Crystal, intensity collection, and refinement data.

	**[Tism$^{Pr^iBenz}$]MgMe•C_6H_6**	**[κ^3-Tism$^{Pr^iBenz}$]ZnMe•C_6H_6**
lattice	Monoclinic	Triclinic
formula	$C_{44}H_{60}N_6Si_3Mg$	$C_{41}H_{57}N_6Si_3Zn$
formula weight	781.56	783.56
space group	$P2_1/n$	P-1
a/Å	12.8340(11)	11.1387(6)
b/Å	17.1903(15)	12.2980(7)
c/Å	20.4108(18)	17.7675(10)
α/°	90	79.0698(9)
β/°	98.1684(13)	75.1020(9)
γ/°	90	65.8782(9)
V/Å^3	4457.4(7)	2136.8(2)
Z	4	2
temperature (K)	130(2)	130(2)
radiation (λ, Å)	0.71073	0.71073
ρ (calcd.) g cm^{-3}	1.165	1.218
μ (Mo Kα), mm^{-1}	0.158	0.694
θ max, deg.	30.596	30.539
no. of data collected	72760	13016
no. of data	13721	13016
no. of parameters	471	474
R_1 [$I > 2\sigma(I)$]	0.0408	0.0602
wR_2 [$I > 2\sigma(I)$]	0.1030	0.1248
R_1 [all data]	0.0527	0.1170
wR_2 [all data]	0.1121	0.1496
GOF	1.025	1.038
R_{int}	0.0333	0.0579

Table 1. (cont.) Crystal, intensity collection, and refinement data.

	**[Tism$^{Pr^iBenz}$]Cu**	**[κ^4-C$_4$-Tism$^{Pr^iBenz*}$]Cu•0.5C_6H_6**
lattice	Cubic	Trigonal
formula	$C_{37}H_{51}CuN_6Si_3$	$C_{80}H_{108}Cu_2N_{12}Si_6$
formula weight	727.64	1533.40
space group	*I23*	*R-3*
a/Å	20.1294(17)	16.4836(19)
b/Å	20.1294(17)	16.4836(19)
c/Å	20.1294(17)	26.069(3)
α/°	90	90
β/°	90	90
γ/°	90	120
V/Å^3	8156(2)	6134.3(16)
Z	8	3
temperature (K)	130(2)	130(2)
radiation (λ, Å)	0.71073	0.71073
ρ (calcd.) g cm^{-3}	1.185	1.245
μ (Mo Kα), mm^{-1}	0.656	0.657
θ max, deg.	30.490	30.548
no. of data collected	66485	33358
no. of data	4176	4192
no. of parameters	145	155
R_1 [$I > 2\sigma(I)$]	0.0511	0.0325
wR_2 [$I > 2\sigma(I)$]	0.1043	0.0861
R_1 [all data]	0.0874	0.0358
wR_2 [all data]	0.1177	0.0903
GOF	1.022	0.960
R_{int}	0.1838	0.0264

Table 1. (cont.) Crystal, intensity collection, and refinement data.

	$[Tism^{Pr^iBenz}]NiBr \cdot THF$	$[Tism^{Pr^iBenz}]NiBr \cdot C_6H_6$
lattice	Triclinic	Monoclinic
formula	$C_{41}H_{59}BrN_6NiOSi_3$	$C_{43}H_{57}BrN_6NiSi_3$
formula weight	874.83	880.83
space group	*P-1*	$P2_1/c$
a / Å	9.4796(9)	12.702(3)
b / Å	15.2993(14)	18.819(5)
c / Å	15.5434(14)	18.915(5)
α / °	89.3810(10)	90
β / °	78.4190(10)	105.630(4)
γ / °	77.8830(10)	90
V / Å^3	2158.0(3)	4354.3(19)
Z	2	4
temperature (K)	130(2)	130(2)
radiation (λ, Å)	0.71073	0.71073
ρ (calcd.) g cm^{-3}	1.346	1.344
μ (Mo Kα), mm^{-1}	1.498	1.483
θ max, deg.	32.918	30.560
no. of data collected	38415	70162
no. of data	15099	13279
no. of parameters	490	499
R_1 [$I > 2\sigma(I)$]	0.0398	0.0620
wR_2 [$I > 2\sigma(I)$]	0.0922	0.1074
R_1 [all data]	0.0618	0.1580
wR_2 [all data]	0.1003	0.1354
GOF	1.057	0.999
R_{int}	0.0316	0.1673

Table 2. Cartesian Coordinates for Geometry Optimized Compounds

[Tism$^{Pr^{i}Benz}$]Li

atom	x	y	z
Li	0.0000000000	0.0000000000	-0.4086697850
Si	-0.8549859254	-1.6017659946	2.0412593996
N	-0.0251719394	-2.0099177932	-0.6470532783
N	-0.7629603951	-3.9674287699	0.1448327903
C	0.0000000000	0.0000000000	1.8730850499
C	-2.7568822645	-1.5107116149	2.1214500123
H	-3.0945305309	-1.1178661697	3.0860016316
H	-3.1944111119	-2.5098443152	2.0094764525
H	-3.1702909094	-0.8801099165	1.3285752701
C	-0.3456847407	-2.6895394153	3.5336815012
H	-1.1386207320	-3.3826333448	3.8392467348
H	-0.1465769778	-2.0356529793	4.3901795433
H	0.5603926074	-3.2764135904	3.3550925350
C	-0.5214623018	-2.6297548560	0.4222601397
C	0.0743371079	-2.9522490683	-1.6521727672
C	0.5452513641	-2.8178972596	-2.9641413901
H	0.9115475891	-1.8594642323	-3.3197670323
C	0.5368100668	-3.9434484665	-3.7780710199
H	0.8970064562	-3.8711660063	-4.8001656989
C	0.0720059864	-5.1832272700	-3.2985446954
H	0.0796111709	-6.0449092060	-3.9599689615

C	-0.3946927838	-5.3347735881	-1.9964236752
H	-0.7434364826	-6.3003134989	-1.6486770521
C	-0.3887617156	-4.1995424893	-1.1749022836
C	-1.3527430617	-4.9434477131	1.0750481201
H	-1.5366551537	-4.3828574773	1.9916255761
C	-0.3656882612	-6.0704587226	1.4101379338
H	0.5690870646	-5.6594904566	1.8020739679
H	-0.1272879328	-6.6802424614	0.5344663556
H	-0.7968749589	-6.7284444005	2.1714570731
C	-2.7067478884	-5.4588390007	0.5663871928
H	-3.3860183550	-4.6249550976	0.3667384201
H	-3.1658760537	-6.1026437801	1.3235571235
H	-2.6063608795	-6.0408036294	-0.3533639769
Si	-0.9596770796	1.5413225286	2.0412593996
N	-1.7280538987	1.0267584356	-0.6470532783
N	-3.0544139049	2.6444574692	0.1448327903
C	0.0701264959	3.1428858838	2.1214500123
H	0.5791647644	3.2388751374	3.0860016316
H	-0.5763833805	4.0213633306	2.0094764525
H	0.8229479089	3.1856074232	1.3285752701
C	-2.1563670878	1.6441414748	3.5336815012
H	-2.3601360423	2.6773911516	3.8392467348
H	-1.6896387045	1.1447658761	4.3901795433
H	-3.1176537063	1.1528925611	3.3550925350
C	-2.0167033602	1.7664770285	0.4222601397
C	-2.5938912454	1.4117467102	-1.6521727672
C	-2.7129962941	0.9367470971	-2.9641413901
H	-2.0661170571	0.1403087472	-3.3197670323

C	-3.6835315839	1.5068330784	-3.7780710199
H	-3.8010313319	1.1587526247	-4.8001656989
C	-4.5248094826	2.5292546215	-3.2985446954
H	-5.2748505214	2.9535093066	-3.9599689615
C	-4.4227030589	3.0092007716	-1.9964236752
H	-5.0845133006	3.7939916295	-1.6486770521
C	-3.4425296222	2.4364487664	-1.1749022836
C	-3.6047797709	3.6432337128	1.0750481201
H	-3.0273383396	3.5222111386	1.9916255761
C	-5.0743273358	3.3519246853	1.4101379338
H	-5.1858060402	2.3369013734	1.8020739679
H	-5.7216157086	3.4503558141	0.5344663556
H	-5.4285662993	4.0543361583	2.1714570731
C	-3.3741193056	5.0735319333	0.5663871928
H	-2.3123194283	5.2448554619	0.3667384201
H	-3.7021065170	5.7930509778	1.3235571235
H	-3.9283089626	5.2775765477	-0.3533639769
Si	1.8146630050	0.0604434660	2.0412593996
N	1.7532258381	0.9831593576	-0.6470532783
N	3.8173743000	1.3229713007	0.1448327903
C	2.6867557686	-1.6321742689	2.1214500123
H	2.5153657665	-2.1210089677	3.0860016316
H	3.7707944924	-1.5115190155	2.0094764525
H	2.3473430005	-2.3054975067	1.3285752701
C	2.5020518284	1.0453979405	3.5336815012
H	3.4987567743	0.7052421932	3.8392467348
H	1.8362156823	0.8908871033	4.3901795433
H	2.5572610989	2.1235210293	3.3550925350

C	2.5381656619	0.8632778276	0.4222601397
C	2.5195541375	1.5405023580	-1.6521727672
C	2.1677449301	1.8811501625	-2.9641413901
H	1.1545694680	1.7191554850	-3.3197670323
C	3.1467215171	2.4366153881	-3.7780710199
H	2.9040248757	2.7124133816	-4.8001656989
C	4.4528034962	2.6539726484	-3.2985446954
H	5.1952393505	3.0913998994	-3.9599689615
C	4.8173958427	2.3255728166	-1.9964236752
H	5.8279497832	2.5063218694	-1.6486770521
C	3.8312913378	1.7630937229	-1.1749022836
C	4.9575228327	1.3002140003	1.0750481201
H	4.5639934933	0.8606463387	1.9916255761
C	5.4400155970	2.7185340373	1.4101379338
H	4.6167189756	3.3225890832	1.8020739679
H	5.8489036414	3.2298866473	0.5344663556
H	6.2254412583	2.6741082422	2.1714570731
C	6.0808671940	0.3853070674	0.5663871928
H	5.6983377834	-0.6199003644	0.3667384201
H	6.8679825707	0.3095928023	1.3235571235
H	6.5346698420	0.7632270817	-0.3533639769

[Tism$^{\mathrm{Pr^iBenz}}$]$^{-}$ (3:0 conformation)

atom	x	y	z
Si	-0.6011214160	-1.7003981850	2.3117914256
N	0.9319953837	-2.0024674719	-0.0745527303
N	-0.6607897850	-3.5848330061	-0.0855958403

C	0.0000000000	0.0000000000	2.4850316125
C	-2.4884476661	-1.8665868969	2.5900452427
H	-2.7235422513	-1.3803410168	3.5423304692
H	-2.8273253589	-2.9042049212	2.6867842929
H	-3.0662939341	-1.3788763153	1.8012621358
C	0.0729311845	-3.0123758995	3.5475033994
H	-0.4848775735	-3.9541215428	3.4497935422
H	-0.0633334725	-2.6548378486	4.5757274611
H	1.1317545720	-3.2437991096	3.4107800568
C	-0.0994333595	-2.4926118045	0.5948660820
C	1.0542396290	-2.7507745256	-1.2249998193
C	1.9690693462	-2.6254105038	-2.2779994958
H	2.7122572830	-1.8343640016	-2.2556213260
C	1.8795385447	-3.5138963137	-3.3451250597
H	2.5753998263	-3.4318161575	-4.1762328931
C	0.8871739295	-4.5120114205	-3.3720957189
H	0.8336524366	-5.1893194538	-4.2212141943
C	-0.0388317138	-4.6477432524	-2.3383255976
H	-0.8028308648	-5.4166826505	-2.3884142056
C	0.0569730993	-3.7576831738	-1.2606762469
C	-1.7773443097	-4.4117618456	0.3783517719
H	-2.0275426382	-4.0196558054	1.3618697531
C	-1.3630322943	-5.8800851920	0.5652723977
H	-0.4992662974	-5.9495910431	1.2332395187
H	-1.0989272360	-6.3603428641	-0.3808672825
H	-2.1893444739	-6.4451053301	1.0110954631
C	-3.0166504568	-4.2461963735	-0.5138280939
H	-3.3062996340	-3.1932133415	-0.5614162534

H	-3.8572526547	-4.8176319880	-0.1038970205
H	-2.8336253748	-4.5985044511	-1.5337667508
Si	-1.1720273168	1.3707855095	2.3117914256
N	-2.2001853928	0.1941020574	-0.0745527303
N	-2.7741615591	2.3646772434	-0.0855958403
C	-0.3722878380	3.0883523433	2.5900452427
H	0.1663607393	3.0488272863	3.5423304692
H	-1.1014525601	3.9006380462	2.6867842929
H	0.3390050494	3.3449266001	1.8012621358
C	-2.6452596469	1.4430276912	3.5475033994
H	-3.1819309190	2.3969770677	3.4497935422
H	-2.2674902836	1.3822673204	4.5757274611
H	-3.3750897197	0.6417713446	3.4107800568
C	-2.1089484647	1.3324177176	0.5948660820
C	-2.9093604338	0.4623889624	-1.2249998193
C	-3.2582068648	-0.3925588237	-2.2779994958
H	-2.9447344667	-1.4317017078	-2.2556213260
C	-3.9828927463	0.1292200297	-3.3451250597
H	-4.2597398866	-0.5144535957	-4.1762328931
C	-4.3511034771	1.4876905498	-3.3720957189
H	-4.9109086936	1.8726955389	-4.2212141943
C	-4.0056478699	2.3575008768	-2.3383255976
H	-4.2895693472	3.4036132491	-2.3884142056
C	-3.2827356375	1.8295014356	-1.2606762469
C	-2.9320256789	3.7451062463	0.3783517719
H	-2.4673527228	3.7657313346	1.3618697531
C	-4.4107870056	4.1204631890	0.5652723977
H	-4.9028638367	3.4071728184	1.2332395187

H	-4.9587548791	4.1318703353	-0.3808672825
H	-4.4869527090	5.1185805971	1.0110954631
C	-2.1689887005	4.7355941167	-0.5138280939
H	-1.1122540565	4.4599461463	-0.5614162534
H	-2.2435653603	5.7492947817	-0.1038970205
H	-2.5656089867	4.7532437850	-1.5337667508
Si	1.7731487328	0.3296126755	2.3117914256
N	1.2681900090	1.8083654145	-0.0745527303
N	3.4349513441	1.2201557627	-0.0855958403
C	2.8607355042	-1.2217654464	2.5900452427
H	2.5571815121	-1.6684862696	3.5423304692
H	3.9287779190	-0.9964331250	2.6867842929
H	2.7272888848	-1.9660502848	1.8012621358
C	2.5723284624	1.5693482082	3.5475033994
H	3.6668084924	1.5571444750	3.4497935422
H	2.3308237561	1.2725705282	4.5757274611
H	2.2433351477	2.6020277650	3.4107800568
C	2.2083818242	1.1601940869	0.5948660820
C	1.8551208047	2.2883855632	-1.2249998193
C	1.2891375186	3.0179693275	-2.2779994958
H	0.2324771837	3.2660657095	-2.2556213260
C	2.1033542016	3.3846762839	-3.3451250597
H	1.6843400603	3.9462697532	-4.1762328931
C	3.4639295476	3.0243208708	-3.3720957189
H	4.0772562570	3.3166239149	-4.2212141943
C	4.0444795837	2.2902423756	-2.3383255976
H	5.0924002119	2.0130694014	-2.3884142056
C	3.2257625383	1.9281817382	-1.2606762469

C	4.7093699886	0.6666555993	0.3783517719
H	4.4948953610	0.2539244707	1.3618697531
C	5.7738192998	1.7596220030	0.5652723977
H	5.4021301342	2.5424182247	1.2332395187
H	6.0576821151	2.2284725288	-0.3808672825
H	6.6762971829	1.3265247330	1.0110954631
C	5.1856391573	-0.4893977432	-0.5138280939
H	4.4185536904	-1.2667328048	-0.5614162534
H	6.1008180150	-0.9316627938	-0.1038970205
H	5.3992343615	-0.1547393338	-1.5337667508

[Tism$^{Pr^iBenz}$]MgMe

**atom**	**x**	**y**	**z**
Mg	3.5298861673	13.7769219536	0.2826572519
N	1.4632843455	14.2703737116	-0.4817126906
N	-0.7390347048	14.6076566209	-0.3034041575
N	4.4602084926	11.8390189659	-0.4176683378
N	4.4473378243	9.6905507958	-1.0362779247
N	4.0716870996	14.5022022475	2.3523476693
N	4.5920143304	14.2628221872	4.5133100807
Si	0.6351280288	13.0889426893	1.9247214881
Si	2.0659985153	10.7162190233	0.5224307565
Si	3.2604611366	11.9283130415	3.1213373415
C	4.6896304376	15.1769169736	-0.8938549006
H	5.6947804802	15.3729469201	-0.4827364943
H	4.2211853658	16.1718754192	-0.9879102883
H	4.8653812153	14.8452181352	-1.9312885877

C	2.1833381779	12.1490481057	1.6565494824
C	-0.9074335086	12.0327247056	2.3356392679
H	-1.7604414466	12.6152383475	2.7025422631
H	-0.6313507805	11.3393628748	3.1392835469
H	-1.2531607755	11.4240589349	1.4936751641
C	0.6667710376	14.4539467516	3.2516272234
H	-0.2856102205	14.9977454980	3.2488352836
H	1.4675750120	15.1754664360	3.0710360508
H	0.7994166797	14.0457985613	4.2597328581
C	0.3850540976	14.0515162078	0.2712961440
C	1.0398708518	14.9822060477	-1.5953648314
C	1.7637683723	15.4474472886	-2.7016029663
H	2.8338007304	15.2885688969	-2.7583470015
C	1.0648127934	16.1216312972	-3.6945648099
H	1.5984574236	16.4952023582	-4.5636948357
C	-0.3244012456	16.3345176790	-3.5976632606
H	-0.8386786524	16.8655168308	-4.3937684653
C	-1.0559947816	15.8799507697	-2.5061603684
H	-2.1247605305	16.0506893266	-2.4507345297
C	-0.3526256972	15.1993047653	-1.5031941852
C	-2.0897284120	14.6028672252	0.2838733133
H	-1.9775371073	14.0868461328	1.2371545803
C	-2.5779695931	16.0274798450	0.5822922011
H	-3.5364839185	15.9862303867	1.1095991518
H	-2.7199497128	16.6134313915	-0.3292098551
H	-1.8597595356	16.5559419882	1.2159173552
C	-3.0732296131	13.7924269195	-0.5719306704
H	-4.0407984795	13.7255510138	-0.0641304736

H	-2.6986130158	12.7772113994	-0.7312345488
H	-3.2372831942	14.2500923432	-1.5510380650
C	1.7205512288	9.0304891127	1.3607739327
H	1.4446021086	8.2332520415	0.6608924546
H	0.8679267000	9.1639447971	2.0375447237
H	2.5570254878	8.6696540660	1.9683458617
C	0.8007675941	10.8317686774	-0.8958402474
H	0.8969945657	9.9585953228	-1.5524358657
H	0.9531914959	11.7280039177	-1.5025937371
H	-0.2310768301	10.8432851599	-0.5279052735
C	3.7733662168	10.6965505181	-0.3750416722
C	5.6312814752	11.5789731581	-1.1158834966
C	6.7056046215	12.4229999611	-1.4273915914
H	6.6826475150	13.4651913750	-1.1330541608
C	7.7726178422	11.8786335797	-2.1304081247
H	8.6194478867	12.5081357425	-2.3879144035
C	7.7796105608	10.5250394497	-2.5201183438
H	8.6315929185	10.1331892727	-3.0684886554
C	6.7211486454	9.6754986211	-2.2189379605
H	6.7461376444	8.6360748039	-2.5245640566
C	5.6433987555	10.2228239482	-1.5100005165
C	3.9512978517	8.3183864998	-1.2360930049
H	2.9701731125	8.3044243905	-0.7617493317
C	3.7509870282	8.0045160697	-2.7256548717
H	3.2865206262	7.0193185560	-2.8358513210
H	4.6956706756	7.9947350372	-3.2750050509
H	3.0963544094	8.7469814661	-3.1912831739
C	4.8334450705	7.2901361830	-0.5144134388

H	4.3889512319	6.2933582150	-0.6001697555
H	4.9213961130	7.5362815874	0.5477293092
H	5.8410024219	7.2473529206	-0.9364827894
C	2.3792938117	11.3141912647	4.7049650182
H	3.0628821514	10.9883001207	5.4974799792
H	1.7759379584	10.4403599839	4.4306639657
H	1.6945587505	12.0530306081	5.1342695836
C	4.7717837578	10.7873397376	2.9225127590
H	5.3800560130	10.8124357792	3.8347770038
H	5.4047943452	11.0960564322	2.0866758344
H	4.4816293043	9.7434581601	2.7600519271
C	4.0283883750	13.6768122002	3.3989525002
C	4.9999995603	15.5453847749	4.1559883742
C	5.6018297141	16.5929510688	4.8670454555
H	5.8577416884	16.5090341414	5.9168281146
C	5.8616474597	17.7672649694	4.1689152424
H	6.3266369341	18.5994590558	4.6899453498
C	5.5358008955	17.9018977670	2.8050977507
H	5.7578958769	18.8358019107	2.2970866239
C	4.9399691068	16.8654089915	2.0988739794
H	4.6964165703	16.9519825973	1.0470790560
C	4.6700310358	15.6759087553	2.7889650934
C	4.7638731175	13.6141104267	5.8243418192
H	4.3629999961	12.6091432263	5.6931364480
C	3.9319455216	14.3098337254	6.9108593972
H	4.0041309923	13.7512720970	7.8496360993
H	2.8788090535	14.3553074698	6.6189197927
H	4.2754689309	15.3305274624	7.0986706573

C	6.2480567529	13.4768807426	6.1930206767
H	6.3468662920	12.9024807385	7.1197588417
H	6.7254140288	14.4479741824	6.3461902848
H	6.7941156031	12.9521425168	5.4037016574

[κ[3]-Tism$^{Pr^iBenz}$]ZnMe

**atom**	**x**	**y**	**z**
Zn	9.4403969630	5.5973893150	14.4902313570
Si	9.8357372679	5.4993957231	11.2671706100
Si	11.9149801748	4.0853299289	13.0980686463
Si	8.9919070494	2.8871858201	12.7714275204
N	8.3111073931	6.9239995182	13.1709075279
N	7.7548818910	7.7062289986	11.1598051912
N	11.4728398336	6.4656302027	14.5786639259
N	13.6783800121	6.2018082552	14.3777277319
N	10.5814851376	1.6728680605	10.7368001210
N	8.8648750184	0.3095337748	11.2364546173
C	10.1229192155	4.3585420877	12.7081080631
C	8.5966605458	5.2640988081	16.3460342014
H	7.5957411312	4.8165740581	16.2878513896
H	8.4923716718	6.2087087908	16.8992233494
H	9.2072331250	4.6030700098	16.9754364204
C	9.2671859359	4.6929885325	9.6373619278
H	9.4796393262	5.3299960499	8.7706987210
H	8.2033282177	4.4362649837	9.6107889524
H	9.8299238598	3.7623423275	9.5100976276
C	11.2878408052	6.6344019199	10.7762211013

H	10.9226853936	7.4408537786	10.1285320432
H	12.0469806410	6.0871713110	10.2091626384
H	11.7697135448	7.1010575724	11.6402895483
C	13.1150483189	3.8155850659	11.6428019811
H	13.9941240207	3.2368609359	11.9513462805
H	13.4671795892	4.7368994899	11.1705057374
H	12.5810180111	3.2184901521	10.8967565645
C	12.2876762762	2.6815423093	14.3240015434
H	13.3488392571	2.6976149369	14.6008753335
H	12.0893412280	1.7077236163	13.8630525935
H	11.7010376328	2.7525953965	15.2439473337
C	7.1872526432	3.3996488141	12.4155143028
H	6.4854795225	2.5686301162	12.5466355438
H	7.0428353694	3.7892817078	11.4030966833
H	6.8863705180	4.1879688045	13.1150852682
C	8.9473821254	1.9981372291	14.4579860465
H	8.0570976657	1.3646918767	14.5455893898
H	8.9039406868	2.7265033988	15.2739731315
H	9.8204034554	1.3608534863	14.6222147560
C	8.5075504037	6.7851616260	11.8609526309
C	7.4059502654	7.9533657042	13.3501717123
C	6.8691462950	8.4863015866	14.5282836536
H	7.1506419267	8.0769926595	15.4935084952
C	5.9687793108	9.5370538902	14.4093707763
H	5.5332348937	9.9747747542	15.3029300882
C	5.6052010514	10.0457757883	13.1468470149
H	4.8973411835	10.8679049461	13.0897615915
C	6.1273590695	9.5218287805	11.9684937169

H	5.8299504796	9.9277718987	11.0084628839
C	7.0393684555	8.4641067542	12.0858283848
C	7.7435591787	7.8651679251	9.6931050806
H	8.4407452669	7.1121789974	9.3246563240
C	8.2765829248	9.2426664854	9.2739985453
H	8.3501756467	9.2933806583	8.1828528979
H	9.2722963778	9.4166603460	9.6927390674
H	7.6244878612	10.0552976178	9.6050044758
C	6.3616536574	7.5464554566	9.1061434823
H	6.4060981137	7.5822890145	8.0128767526
H	5.6011914052	8.2596636773	9.4360588990
H	6.0385121583	6.5443950007	9.4029793222
C	12.4325002508	5.6963757747	14.0623163359
C	13.4907412623	7.3587701967	15.1327507741
C	14.3674201547	8.2830136718	15.7174961516
H	15.4445579468	8.1912984516	15.6364842946
C	13.8018969204	9.3429103353	16.4197414031
H	14.4564037381	10.0745511440	16.8852097080
C	12.4057205880	9.4908100366	16.5423791647
H	12.0075070129	10.3327760237	17.1007780826
C	11.5359021407	8.5771776707	15.9603610778
H	10.4587811528	8.6804376872	16.0472012248
C	12.0908513560	7.5048108726	15.2506135556
C	14.9667634106	5.5868173941	14.0065274619
H	14.6981432140	4.6931713032	13.4423150801
C	15.7767950158	6.4987913749	13.0745188793
H	16.6793430695	5.9776875066	12.7391375693
H	16.0869003758	7.4227602752	13.5707059859

H	15.1895904028	6.7680496372	12.1916670454
C	15.7529663750	5.1387871389	15.2469766099
H	16.6545458774	4.6001833533	14.9373432596
H	15.1492965784	4.4685748378	15.8661202216
H	16.0628990734	5.9848287035	15.8664169045
C	9.4971459672	1.5305022168	11.4874759903
C	10.6796564943	0.5294162160	9.9675066283
C	11.6407583853	0.1790479383	9.0096951086
H	12.4608569771	0.8557280929	8.7881623110
C	11.5087417426	-1.0438840202	8.3643166326
H	12.2393355912	-1.3415925570	7.6171544676
C	10.4376858711	-1.9096062971	8.6622919284
H	10.3610332906	-2.8593285614	8.1397318080
C	9.4734013584	-1.5797308148	9.6098008807
H	8.6582900646	-2.2629879105	9.8203908389
C	9.6074882993	-0.3460171880	10.2624704688
C	7.6498549646	-0.1967643316	11.8955427356
H	7.3674195553	0.5790971100	12.6078384785
C	7.9345563689	-1.4754821381	12.6974156292
H	7.0369984967	-1.7785142529	13.2466941854
H	8.7372297116	-1.3041571985	13.4205416230
H	8.2313859949	-2.3069637382	12.0524969839
C	6.4921194740	-0.3539045674	10.8988030361
H	5.5810654607	-0.6525945757	11.4280305533
H	6.7050746805	-1.1121863444	10.1403885449
H	6.2962676968	0.5922437948	10.3855766161

[κ^3-Tism$^{Pr^iBenz}$]MgMe

atom	x	y	z
Mg	9.4792598954	5.6653873854	14.4723383483
Si	9.8418319355	5.5019908375	11.2655511415
Si	11.9308371826	4.0973070233	13.0905253769
Si	9.0097234063	2.8965926623	12.7678118735
N	8.3153722457	6.9181967611	13.1865524988
N	7.7432373484	7.6965583204	11.1791493067
N	11.4700476450	6.4925316432	14.5509048622
N	13.6764058245	6.2242921270	14.3762628465
N	10.6061648756	1.6531966640	10.7539051842
N	8.8460602986	0.3328165981	11.2159442597
C	10.1436604777	4.3618816730	12.6973189855
C	8.6158900143	5.2952495513	16.3816747681
H	7.6057619356	4.8599517358	16.3516601408
H	8.5167849778	6.2325474368	16.9542856009
H	9.2108569039	4.6288584709	17.0244824207
C	9.2705425378	4.6964983411	9.6367716462
H	9.4722165640	5.3368926629	8.7705130176
H	8.2105563365	4.4256002160	9.6145684053
H	9.8456138421	3.7738348839	9.5074835552
C	11.2812010558	6.6455734512	10.7592840614
H	10.9045772519	7.4467888265	10.1123218892
H	12.0353637317	6.0984822030	10.1858018851
H	11.7709078222	7.1179387749	11.6153330561
C	13.1374924790	3.8273620076	11.6422287315

H	14.0307959080	3.2759008718	11.9583833080
H	13.4652629077	4.7466483959	11.1495232523
H	12.6124729678	3.2031775133	10.9121843013
C	12.3119318696	2.7081993586	14.3297586532
H	13.3737293874	2.7315251025	14.6018884285
H	12.1148350882	1.7294518867	13.8794648610
H	11.7281059844	2.7876184165	15.2507251931
C	7.2053371598	3.4165016855	12.4127164779
H	6.4955464790	2.6034792034	12.5978471644
H	7.0499627568	3.7555697301	11.3839907791
H	6.9248102285	4.2422182586	13.0762081751
C	8.9643375703	2.0183501224	14.4572484673
H	8.0988226407	1.3498660756	14.5294409674
H	8.8735274810	2.7513075503	15.2660240139
H	9.8585795343	1.4185665332	14.6443657383
C	8.5088968950	6.7835478856	11.8727778386
C	7.3949024021	7.9356347865	13.3700774990
C	6.8508976632	8.4551194247	14.5516520217
H	7.1349455630	8.0414343210	15.5141310158
C	5.9374761281	9.4956440585	14.4384328376
H	5.4952773165	9.9226136189	15.3333214589
C	5.5692472406	10.0050936653	13.1770912361
H	4.8518969593	10.8187468807	13.1224575568
C	6.0967106489	9.4919774184	11.9962101209
H	5.7943031765	9.8978604848	11.0380571603
C	7.0208907944	8.4445617874	12.1083402570
C	7.7256686733	7.8527149681	9.7128289795
H	8.4274938501	7.1045832214	9.3445578539

C	8.2462280669	9.2332024259	9.2883768283
H	8.3202994168	9.2787042218	8.1972373987
H	9.2396939815	9.4183031690	9.7072172065
H	7.5856828870	10.0408038418	9.6143393280
C	6.3452825557	7.5217042241	9.1295531254
H	6.3893812995	7.5525001673	8.0363840550
H	5.5806515660	8.2317275950	9.4559151015
H	6.0294385113	6.5190451131	9.4314375902
C	12.4369647284	5.7173176091	14.0495193988
C	13.4826799248	7.3874192166	15.1198223497
C	14.3547384748	8.3159618156	15.7035517480
H	15.4319883383	8.2208520307	15.6348511063
C	13.7838426422	9.3841511554	16.3880746440
H	14.4342449071	10.1199879563	16.8517636136
C	12.3870419842	9.5355211356	16.4947640231
H	11.9844429308	10.3840048344	17.0393459985
C	11.5217059427	8.6167289357	15.9146779654
H	10.4442189947	8.7228783657	15.9908802611
C	12.0831782054	7.5366380905	15.2221289322
C	14.9665796800	5.6015437206	14.0261299038
H	14.7002297995	4.7060697247	13.4643649096
C	15.7922988214	6.5028250258	13.0983623550
H	16.6963417703	5.9746110296	12.7798286475
H	16.0999838289	7.4290974152	13.5910405245
H	15.2178584932	6.7664595517	12.2057431875
C	15.7324887603	5.1584833105	15.2804001360
H	16.6336440594	4.6108040658	14.9869914573
H	15.1151951730	4.4982379149	15.8965060532

H	16.0403083238	6.0076124625	15.8962472951
C	9.5049427719	1.5356448945	11.4841484683
C	10.6882902567	0.5108049925	9.9816711067
C	11.6577219100	0.1383372629	9.0414284311
H	12.4998171156	0.7931991261	8.8392716236
C	11.5056753086	-1.0779623257	8.3881291123
H	12.2424425376	-1.3927064453	7.6544918513
C	10.4060765018	-1.9155695298	8.6606591906
H	10.3141440377	-2.8605021026	8.1323253961
C	9.4328418477	-1.5638151017	9.5907959783
H	8.5955442705	-2.2252309418	9.7825155955
C	9.5879781447	-0.3372560831	10.2520159191
C	7.6055471244	-0.1416664127	11.8499798789
H	7.3361629723	0.6363428806	12.5645846752
C	7.8360824441	-1.4352410013	12.6445784717
H	6.9201801726	-1.7126833505	13.1763199177
H	8.6316956547	-1.2954977384	13.3817777340
H	8.1158703448	-2.2705503106	11.9973805202
C	6.4604931898	-0.2535675597	10.8328926625
H	5.5313923110	-0.5250986584	11.3446259412
H	6.6614328028	-1.0136797808	10.0732741088
H	6.3052355332	0.7018561924	10.3233828440

[Tism$^{\mathrm{Pr^iBenz}}$]ZnMe

atom	x	y	z
Zn	3.5455907281	13.7733655362	0.2823311321
N	1.3916997648	14.2610358675	-0.5905675201

N	-0.8070028344	14.5867138555	-0.3404869746
N	4.5915240948	11.7588434392	-0.4105153474
N	4.4972246075	9.6146651124	-1.0403959219
N	4.0735210493	14.5962379448	2.4342912556
N	4.5772629137	14.2938327272	4.5922305245
Si	0.6672537426	13.1128699561	1.8544129899
Si	2.1473074912	10.7428078566	0.4977998671
Si	3.2818769130	11.9980236254	3.1021539028
C	4.6585395731	15.1116040160	-0.8624056057
H	5.6570005053	15.2852965964	-0.4357896563
H	4.1692714626	16.0932572952	-0.9398560597
H	4.8095869383	14.7482317851	-1.8891202647
C	2.2604136107	12.2264085372	1.5852042979
C	-0.8184446579	11.9932187781	2.3021523177
H	-1.6993849467	12.5471298226	2.6467014637
H	-0.5173901492	11.3382543692	3.1285442548
H	-1.1338522659	11.3450087549	1.4776284924
C	0.6716441577	14.4980219374	3.1614188438
H	-0.3006822867	15.0060781752	3.1736853809
H	1.4408030090	15.2453828062	2.9492629906
H	0.8463814047	14.1161822222	4.1735289694
C	0.3439239775	14.0376203467	0.1959839870
C	0.9309132646	14.9725764801	-1.6852192780
C	1.6224000437	15.4502237082	-2.8063806994
H	2.6917221724	15.2930885357	-2.8949583361
C	0.8946983370	16.1306752785	-3.7741767880
H	1.4018648918	16.5148105929	-4.6547181505
C	-0.4924041527	16.3356749513	-3.6363867389

H	-1.0303655988	16.8716051654	-4.4135617947
C	-1.1930057419	15.8695391979	-2.5287898603
H	-2.2608270271	16.0371606275	-2.4443598094
C	-0.4605014258	15.1843014567	-1.5497460453
C	-2.1353968641	14.5844097400	0.2950077457
H	-1.9928155734	14.0619469368	1.2414910923
C	-2.6059713553	16.0100099409	0.6177787319
H	-3.5486693993	15.9724771066	1.1735376367
H	-2.7710660468	16.6027140326	-0.2857728312
H	-1.8654208676	16.5301323958	1.2325740312
C	-3.1537947079	13.7845695493	-0.5297216817
H	-4.1050962606	13.7241129121	0.0091653371
H	-2.7934882124	12.7665129411	-0.7045538624
H	-3.3464268243	14.2466866865	-1.5018244295
C	1.7616712795	9.1023977040	1.4041807349
H	1.5205533332	8.2717523336	0.7308224504
H	0.8793526599	9.2592606586	2.0362338889
H	2.5731852814	8.7730723656	2.0617941156
C	0.9047855912	10.8334072394	-0.9426237818
H	0.9852106083	9.9346551236	-1.5667142627
H	1.0921338667	11.7035232252	-1.5773691468
H	-0.1328341174	10.8902447199	-0.5951460109
C	3.8643933133	10.6469628615	-0.3709164405
C	5.7435658645	11.4634048765	-1.1192467021
C	6.8375083106	12.2775115753	-1.4414105488
H	6.8500948936	13.3187213077	-1.1384529731
C	7.8786532093	11.7072948939	-2.1625961918
H	8.7404735962	12.3124750423	-2.4294153284

C	7.8392405053	10.3557310601	-2.5586801217
H	8.6717145300	9.9420161572	-3.1212759028
C	6.7597836047	9.5350862184	-2.2483582892
H	6.7512487477	8.4973170568	-2.5619495552
C	5.7069350933	10.1087512332	-1.5223722511
C	3.9462055678	8.2654610312	-1.2521203227
H	2.9702675085	8.2817557064	-0.7658256949
C	3.7167290214	7.9797509381	-2.7433861841
H	3.2181710060	7.0122110011	-2.8630690269
H	4.6542618919	7.9471230858	-3.3045436921
H	3.0819374111	8.7507031572	-3.1901770353
C	4.7959017626	7.1929288474	-0.5558440146
H	4.3143246818	6.2143305587	-0.6532057776
H	4.9032173934	7.4177424880	0.5094156912
H	5.7974059390	7.1196544087	-0.9886263311
C	2.3301499286	11.3459204989	4.6287074086
H	2.9805707161	11.0413073719	5.4568237865
H	1.7699479428	10.4530478545	4.3261298980
H	1.6021122683	12.0629081519	5.0229120435
C	4.8136571917	10.8779002162	2.9416611071
H	5.3851946204	10.8860470025	3.8782032372
H	5.4740229963	11.2174033196	2.1392641928
H	4.5481912814	9.8346574062	2.7372242639
C	4.0180158415	13.7438568576	3.4529651556
C	5.0007987655	15.5809601170	4.2704987537
C	5.6135074763	16.6041426058	5.0067960610
H	5.8633814698	16.4950648441	6.0561547695
C	5.8950543220	17.7891915482	4.3351570585

H	6.3704308256	18.6022687763	4.8769164248
C	5.5802198191	17.9599272842	2.9726092449
H	5.8211108879	18.9015615644	2.4874877898
C	4.9712921998	16.9483808009	2.2412590037
H	4.7317205185	17.0624129900	1.1897740215
C	4.6791123358	15.7491111250	2.9040689099
C	4.7409473554	13.6062764192	5.8840427945
H	4.3259767117	12.6103326201	5.7259189248
C	3.9198555762	14.2826567647	6.9909665977
H	3.9881505295	13.6995703314	7.9152799181
H	2.8662108351	14.3478757440	6.7038526275
H	4.2754969827	15.2944329328	7.2041584102
C	6.2234914103	13.4392380060	6.2475973816
H	6.3162532737	12.8419579346	7.1606446942
H	6.7145266370	14.4001208453	6.4224167091
H	6.7615031242	12.9256383898	5.4452323839

[Tism$^{\mathrm{Pr^iBenz}}$]Cu

**atom**	**x**	**y**	**z**
Cu	0.0000000000	0.0000000000	-0.3009274257
Si	-0.8609484707	-1.6190936444	2.0826040831
N	-0.0255567286	-2.0536158436	-0.5862178328
N	-0.7495107080	-4.0196129898	0.1796852305
C	0.0000000000	0.0000000000	1.9297532225
C	-2.7637556939	-1.5105033081	2.1085226092
H	-3.1321943655	-1.1000068726	3.0543878751
H	-3.2055590033	-2.5079937566	1.9957894460

H	-3.1422944895	-0.8886451132	1.2913691353
C	-0.3833915694	-2.6941920239	3.5923877328
H	-1.1610105985	-3.4230951902	3.8512632543
H	-0.2500141762	-2.0448555168	4.4652213586
H	0.5521707724	-3.2440108734	3.4486022948
C	-0.5126745637	-2.6855323085	0.4789356859
C	0.0711230270	-2.9728173789	-1.6112346308
C	0.5239339111	-2.8086764416	-2.9258859063
H	0.8753736393	-1.8383821323	-3.2642668780
C	0.5090944904	-3.9187295142	-3.7611195064
H	0.8535347967	-3.8258409268	-4.7874028168
C	0.0551401844	-5.1695313222	-3.2978535185
H	0.0563711047	-6.0187728905	-3.9757469735
C	-0.3946453673	-5.3478635432	-1.9927895951
H	-0.7364618341	-6.3219653350	-1.6619450876
C	-0.3828815078	-4.2284844284	-1.1482966581
C	-1.3484912155	-5.0118666189	1.0866845657
H	-1.5348108968	-4.4702104615	2.0150162680
C	-0.3682365166	-6.1515989866	1.3971028802
H	0.5647427704	-5.7555396473	1.8082544042
H	-0.1243270148	-6.7342642306	0.5044388374
H	-0.8072026679	-6.8320479994	2.1339201204
C	-2.7004607049	-5.5093177751	0.5542685577
H	-3.3736571741	-4.6676951962	0.3666624216
H	-3.1707439828	-6.1691466136	1.2904770081
H	-2.5928982579	-6.0691517192	-0.3785935982
Si	-0.9717019918	1.5551500692	2.0826040831
N	-1.7657051258	1.0489406980	-0.5862178328

N	-3.1063316086	2.6589018085	0.1796852305
C	0.0737436096	3.1487342948	2.1085226092
H	0.6134632867	3.2625633265	3.0543878751
H	-0.5692068041	4.0300924085	1.9957894460
H	0.8015580018	3.1656294106	1.2913691353
C	-2.1415429507	1.6791228506	3.5923877328
H	-2.3839820950	2.7170122675	3.8512632543
H	-1.6458897366	1.2389463863	4.4652213586
H	-3.0854812127	1.1438115206	3.4486022948
C	-2.0694019200	1.7867553503	0.4789356859
C	-2.6100968844	1.4248143413	-1.6112346308
C	-2.6943521049	0.9505981439	-2.9258859063
H	-2.0297724481	0.1610952567	-3.2642668780
C	-3.6482665551	1.5184759955	-3.7611195064
H	-3.7400428318	1.1737376465	-4.7874028168
C	-4.5045155429	2.5370128607	-3.2978535185
H	-5.2405957751	2.9605676365	-3.9757469735
C	-4.4340630007	3.0157046852	-1.9927895951
H	-5.1067516649	3.7987773247	-1.6619450876
C	-3.4705341806	2.4458273266	-1.1482966581
C	-3.6661582046	3.6737609589	1.0866845657
H	-3.1039103715	3.5642904574	2.0150162680
C	-5.1433227380	3.3947016712	1.3971028802
H	-5.2668149323	2.3886882379	1.8082544042
H	-5.7698803921	3.4748024685	0.5044388374
H	-5.5131257934	4.1150820161	2.1339201204
C	-3.4209787983	5.0933264599	0.5542685577
H	-2.3555140300	5.2555204145	0.3666624216

H	-3.7572656956	5.8305181448	1.2904770081
H	-3.9595904393	5.2800916203	-0.3785935982
Si	1.8326504625	0.0639435752	2.0826040831
N	1.7912618545	1.0046751456	-0.5862178328
N	3.8558423166	1.3607111814	0.1796852305
C	2.6900120843	-1.6382309867	2.1085226092
H	2.5187310788	-2.1625564538	3.0543878751
H	3.7747658074	-1.5220986519	1.9957894460
H	2.3407364877	-2.2769842975	1.2913691353
C	2.5249345201	1.0150691733	3.5923877328
H	3.5449926935	0.7060829227	3.8512632543
H	1.8959039127	0.8059091306	4.4652213586
H	2.5333104403	2.1001993528	3.4486022948
C	2.5820764837	0.8987769582	0.4789356859
C	2.5389738574	1.5480030376	-1.6112346308
C	2.1704181939	1.8580782977	-2.9258859063
H	1.1543988088	1.6772868756	-3.2642668780
C	3.1391720647	2.4002535187	-3.7611195064
H	2.8865080352	2.6521032803	-4.7874028168
C	4.4493753585	2.6325184616	-3.2978535185
H	5.1842246704	3.0582052540	-3.9757469735
C	4.8287083680	2.3321588580	-1.9927895951
H	5.8432134990	2.5231880103	-1.6619450876
C	3.8534156884	1.7826571018	-1.1482966581
C	5.0146494201	1.3381056600	1.0866845657
H	4.6387212683	0.9059200041	2.0150162680
C	5.5115592545	2.7568973154	1.3971028802
H	4.7020721619	3.3668514094	1.8082544042

H	5.8942074069	3.2594617621	0.5044388374
H	6.3203284613	2.7169659833	2.1339201204
C	6.1214395032	0.4159913152	0.5542685577
H	5.7291712040	-0.5878252183	0.3666624216
H	6.9280096784	0.3386284687	1.2904770081
H	6.5524886971	0.7890600988	-0.3785935982

[κ^4-C_4-Tism$^{Pr^iBenz*}$]Cu

atom	x	y	z
Cu	0.0000000000	0.0000000000	-0.4034690839
Si	1.7303448966	-0.5441133610	2.2908545411
N	2.6808569520	-0.0738508346	0.7566904387
N	2.9365828164	0.3679894382	-1.3804492676
C	0.0000000000	0.0000000000	2.1421419594
C	2.7570192394	0.2038221556	3.7134864035
H	3.2008720910	1.1720921266	3.4636754807
H	3.5668701391	-0.4659644596	4.0260986970
H	2.1098890652	0.3523112665	4.5850037882
C	1.9998579887	-2.4196274245	2.3713850781
H	1.4095296880	-2.9517374366	1.6190560542
H	1.7423572160	-2.8234102513	3.3561707698
H	3.0568141100	-2.6474422006	2.1888948915
C	2.0007925839	0.1344565718	-0.4113409087
C	4.0601042200	0.0034871238	0.5176261610
C	5.1707401424	-0.1618095021	1.3499317909
H	5.0555194739	-0.3889574353	2.4025543339
C	6.4381982873	-0.0242473973	0.7861671899

H	7.3153867448	-0.1501229874	1.4143683808
C	6.6012078573	0.2727969532	-0.5745620277
H	7.6015652981	0.3753178110	-0.9852524435
C	5.4996728575	0.4360810190	-1.4132709099
H	5.6381644857	0.6616814040	-2.4644004647
C	4.2285004964	0.2933966013	-0.8507016852
C	2.5573891304	0.6695760311	-2.7686997557
H	1.4639792069	0.6387942211	-2.7424848098
C	2.9849105114	2.0863732885	-3.1752698494
H	2.5808139778	2.8230417810	-2.4743067829
H	2.6053692608	2.3185437291	-4.1761517990
H	4.0727601061	2.1997144832	-3.1963211347
C	3.0483659628	-0.4117055697	-3.7407967278
H	2.6954817252	-1.3987576419	-3.4271851687
H	4.1398305722	-0.4476708941	-3.8030486039
H	2.6637189109	-0.2114139783	-4.7467227690
Si	-0.3939564551	1.7705793183	2.2908545411
N	-1.2764717771	2.3586156416	0.7566904387
N	-1.7869796100	2.3591606002	-1.3804492676
C	-1.5550247843	2.2857376222	3.7134864035
H	-2.6154976027	2.1859904818	3.4636754807
H	-1.3798980103	3.3219823822	4.0260986970
H	-1.3600550394	1.6510618963	4.5850037882
C	1.0955298230	2.9417415344	2.3713850781
H	1.8515147614	2.6965572354	1.6190560542
H	1.5739663949	2.9206307372	3.3561707698
H	0.7643451458	3.9709997742	2.1888948915
C	-1.1168390988	1.6655089194	-0.4113409087

C	-2.0330720478	3.5144098346	0.5176261610
C	-2.4452389318	4.5588970707	1.3499317909
H	-2.1909127170	4.5726870113	2.4025543339
C	-3.1981002817	5.5877669700	0.7861671899
H	-3.5276830516	6.4103722532	1.4143683808
C	-3.5368530202	5.5804152235	-0.5745620277
H	-4.1258174079	6.3954897512	-0.9852524435
C	-3.1274936693	4.5448158976	-1.4132709099
H	-3.3921151479	4.5519529733	-2.4644004647
C	-2.3683391583	3.5152905491	-0.8507016852
C	-1.8585644179	1.8799759387	-2.7686997557
H	-1.2852016268	0.9484460733	-2.7424848098
C	-3.2993075253	1.5418216867	-3.1752698494
H	-3.7352328872	0.8235295767	-2.4743067829
H	-3.3106023996	1.0970441015	-4.1761517990
H	-3.9413886766	2.4272564738	-3.1963211347
C	-1.1676354992	2.8458151486	-3.7407967278
H	-0.1363812110	3.0337344704	-3.4271851687
H	-1.6822209192	3.8090338899	-3.8030486039
H	-1.1487695795	2.4125552346	-4.7467227690
Si	-1.3363884415	-1.2264659573	2.2908545411
N	-1.4043851748	-2.2847648070	0.7566904387
N	-1.1496032064	-2.7271500384	-1.3804492676
C	-1.2019944551	-2.4895597778	3.7134864035
H	-0.5853744883	-3.3580826083	3.4636754807
H	-2.1869721288	-2.8560179227	4.0260986970
H	-0.7498340257	-2.0033731628	4.5850037882
C	-3.0953878117	-0.5221141099	2.3713850781

H	-3.2610444493	0.2551802011	1.6190560542
H	-3.3163236110	-0.0972204859	3.3561707698
H	-3.8211592558	-1.3235575736	2.1888948915
C	-0.8839534850	-1.7999654912	-0.4113409087
C	-2.0270321722	-3.5178969584	0.5176261610
C	-2.7255012106	-4.3970875686	1.3499317909
H	-2.8646067569	-4.1837295760	2.4025543339
C	-3.2400980057	-5.5635195728	0.7861671899
H	-3.7877036931	-6.2602492658	1.4143683808
C	-3.0643548371	-5.8532121767	-0.5745620277
H	-3.4757478902	-6.7708075622	-0.9852524435
C	-2.3721791882	-4.9808969166	-1.4132709099
H	-2.2460493378	-5.2136343773	-2.4644004647
C	-1.8601613381	-3.8086871504	-0.8507016852
C	-0.6988247125	-2.5495519698	-2.7686997557
H	-0.1787775802	-1.5872402944	-2.7424848098
C	0.3143970139	-3.6281949752	-3.1752698494
H	1.1544189094	-3.6465713578	-2.4743067829
H	0.7052331388	-3.4155878306	-4.1761517990
H	-0.1313714295	-4.6269709570	-3.1963211347
C	-1.8807304636	-2.4341095790	-3.7407967278
H	-2.5591005142	-1.6349768285	-3.4271851687
H	-2.4576096529	-3.3613629958	-3.8030486039
H	-1.5149493314	-2.2011412562	-4.7467227690

[κ⁴-C_4-Tism$^{Pr^iBenz*}$]Li

atom	x	y	z
Li	0.0000000000	0.0000000000	-0.3300570047
Si	1.7260091104	-0.5420331653	2.1777926781
N	2.7197547625	-0.0646429335	0.6785080933
N	3.0607493550	0.5018115247	-1.4087789279
C	0.0000000000	0.0000000000	1.9768074541
C	2.7133684221	0.2060347425	3.6275575109
H	3.1640878760	1.1724929300	3.3838388567
H	3.5153587489	-0.4623970567	3.9611455400
H	2.0468080381	0.3576428507	4.4835202962
C	2.0031385539	-2.4156696156	2.2643789525
H	1.4196110993	-2.9542335250	1.5117887583
H	1.7444938336	-2.8166748516	3.2496151973
H	3.0617890457	-2.6381378888	2.0866286367
C	2.0819479705	0.2495357360	-0.4896955163
C	4.1099318493	-0.0368719769	0.4872773870
C	5.1852671270	-0.3079308473	1.3391414662
H	5.0255587401	-0.5990133081	2.3698366526
C	6.4751117922	-0.1916395768	0.8249254605
H	7.3255705166	-0.3981398909	1.4677592284
C	6.6928665019	0.1879860388	-0.5085685679
H	7.7089801815	0.2724687055	-0.8827413202
C	5.6275681678	0.4564168058	-1.3657963085
H	5.8098006221	0.7451934143	-2.3944557434
C	4.3328940280	0.3346902570	-0.8523761782
C	2.7348034954	0.8983316656	-2.7873192790

H	1.6413157221	0.9145460262	-2.7939070510
C	3.2327103910	2.3151179650	-3.1036766780
H	2.8473328322	3.0288358868	-2.3696972366
H	2.8845539717	2.6198424213	-4.0962086616
H	4.3246082337	2.3792824269	-3.0992016461
C	3.2062857389	-0.1465632751	-3.8075800432
H	2.8073976522	-1.1341007844	-3.5572693827
H	4.2964724692	-0.2231340493	-3.8465146175
H	2.8546785550	0.1237058786	-4.8088569067
Si	-0.3935900644	1.7657843194	2.1777926781
N	-1.3038949587	2.3876981832	0.6785080933
N	-1.9649562058	2.3997809337	-1.4087789279
C	-1.5351155322	2.2468286121	3.6275575109
H	-2.5974526012	2.1539340155	3.3838388567
H	-1.3572317768	3.2755885083	3.9611455400
H	-1.3331318132	1.5937663324	4.4835202962
C	1.0904619773	2.9426036827	2.2643789525
H	1.8486357317	2.7065360380	1.5117887583
H	1.5670650589	2.9191134024	3.2496151973
H	0.7537999076	3.9706560390	2.0866286367
C	-1.2570782718	1.6782519638	-0.4896955163
C	-2.0230338560	3.5777413778	0.4872773870
C	-2.3259576271	4.6445384810	1.3391414662
H	-1.9940186280	4.6517681912	2.3698366526
C	-3.0715911542	5.7034310928	0.8249254605
H	-3.3179859985	6.5432001100	1.4677592284
C	-3.5092339361	5.7021993954	-0.5085685679
H	-4.0904549114	6.5399383217	-0.8827413202

C	-3.2090526325	4.6454085919	-1.3657963085
H	-3.5502567386	4.6588382225	-2.3944557434
C	-2.4562972790	3.5850511717	-0.8523761782
C	-2.1453797912	1.9192434686	-2.7873192790
H	-1.6126779527	0.9641480979	-2.7939070510
C	-3.6213061660	1.6420503392	-3.1036766780
H	-4.0467152380	0.9514446223	-2.3696972366
H	-3.7111270766	1.1881758074	-4.0962086616
H	-4.2228231413	2.5555793783	-3.0992016461
C	-1.4762153500	2.8500065393	-3.8075800432
H	-0.4215387364	2.9983280775	-3.5572693827
H	-1.9549964795	3.8324213296	-3.8465146175
H	-1.5344717110	2.4103712090	-4.8088569067
Si	-1.3324190460	-1.2237511542	2.1777926781
N	-1.4158598038	-2.3230552497	0.6785080933
N	-1.0957931492	-2.9015924584	-1.4087789279
C	-1.1782528900	-2.4528633547	3.6275575109
H	-0.5666352749	-3.3264269455	3.3838388567
H	-2.1581269722	-2.8131914517	3.9611455400
H	-0.7136762249	-1.9514091830	4.4835202962
C	-3.0936005312	-0.5269340672	2.2643789525
H	-3.2682468310	0.2476974870	1.5117887583
H	-3.3115588925	-0.1024385509	3.2496151973
H	-3.8155889533	-1.3325181502	2.0866286367
C	-0.8248696987	-1.9277876999	-0.4896955163
C	-2.0868979933	-3.5408694009	0.4872773870
C	-2.8593094999	-4.3366076337	1.3391414662
H	-3.0315401121	-4.0527548831	2.3698366526

C	-3.4035206380	-5.5117915160	0.8249254605
H	-4.0075845181	-6.1450602191	1.4677592284
C	-3.1836325658	-5.8901854341	-0.5085685679
H	-3.6185252701	-6.8124070272	-0.8827413202
C	-2.4185155353	-5.1018253978	-1.3657963085
H	-2.2595438835	-5.4040316368	-2.3944557434
C	-1.8765967491	-3.9197414287	-0.8523761782
C	-0.5894237042	-2.8175751342	-2.7873192790
H	-0.0286377694	-1.8786941241	-2.7939070510
C	0.3885957750	-3.9571683042	-3.1036766780
H	1.1993824058	-3.9802805092	-2.3696972366
H	0.8265731049	-3.8080182287	-4.0962086616
H	-0.1017850924	-4.9348618052	-3.0992016461
C	-1.7300703890	-2.7034432641	-3.8075800432
H	-2.3858589158	-1.8642272931	-3.5572693827
H	-2.3414759897	-3.6092872803	-3.8465146175
H	-1.3202068440	-2.5340770876	-4.8088569067

[Tism$^{Pr^iBenz*}$]$^{-}$ (3:0 conformation)

atom	x	y	z
Si	1.7475084390	0.2267462312	2.2881051725
N	2.5348108784	0.8287795370	0.6889447303
N	3.2906358706	2.3291848572	-0.7102519840
C	-0.0501606390	0.0345754568	2.2611283268
C	2.4868881543	1.4845439550	3.5113629447
H	2.2365367229	2.5170275498	3.2671906870
H	3.5809143385	1.3980926715	3.5101437695

H	2.1331614549	1.2676656930	4.5265906481
C	2.7314649568	-1.3546263285	2.6802888026
H	2.3882598622	-2.2514844746	2.1560977179
H	2.6361663321	-1.5451187906	3.7559007214
H	3.7972022200	-1.2070905840	2.4693300291
C	2.5108658538	2.1702041833	0.4153713501
C	3.3012923206	0.1452561901	-0.2651577025
C	3.5925883990	-1.2099903274	-0.4562850404
H	3.1628576306	-1.9720748464	0.1809484586
C	4.4356098096	-1.5583134367	-1.5115754967
H	4.6855508104	-2.6045630615	-1.6663899630
C	4.9668345780	-0.5890847292	-2.3752649422
H	5.6216770703	-0.8926714135	-3.1881892824
C	4.6563225490	0.7601482709	-2.2128828899
H	5.0616683135	1.5018195364	-2.8922318432
C	3.8117049870	1.1136334672	-1.1558102635
C	3.5484138626	3.6599575009	-1.2660065634
H	2.8918824743	4.3053576145	-0.6755598654
C	5.0015150020	4.0956819334	-1.0245106139
H	5.2343875545	4.0586869686	0.0435726375
H	5.1575916608	5.1210993839	-1.3785971977
H	5.7090105162	3.4456283754	-1.5494274186
C	3.1419598128	3.7720204180	-2.7426488212
H	2.1315939740	3.3840006727	-2.8931491861
H	3.8153430391	3.2175842655	-3.4026182455
H	3.1610984231	4.8217745901	-3.0560304063
Si	-1.1515140234	1.4683285028	2.4008134227
N	-2.2925194450	1.7198675608	0.9413201191

N	-4.1531417274	1.7476123065	-0.2015622213
C	-2.3859858394	1.4448155290	3.8451913517
H	-3.0547858484	0.5831062469	3.8210333712
H	-3.0087434980	2.3466815446	3.8471069218
H	-1.8166386785	1.4162794956	4.7830483890
C	-0.3153896895	3.1715917140	2.5356177017
H	0.5484881303	3.2919714982	1.8758932507
H	0.0236295563	3.3284701997	3.5660180167
H	-1.0458471088	3.9571881964	2.3073589630
C	-3.6142247180	1.3658969936	1.0100848057
C	-2.0001014468	2.3148419604	-0.2949640938
C	-0.8160883997	2.8269425183	-0.8343067871
H	0.1217967393	2.7807531322	-0.2884924361
C	-0.8732573226	3.3850087129	-2.1117701351
H	0.0284063241	3.8024786934	-2.5500054368
C	-2.0704982455	3.4223620258	-2.8423732938
H	-2.0796048128	3.8608449877	-3.8373206591
C	-3.2511478120	2.8991089691	-2.3154424739
H	-4.1700158044	2.9260827661	-2.8915436968
C	-3.2012535901	2.3448882364	-1.0323868605
C	-5.5753177518	1.5408861309	-0.4804937126
H	-5.9328708958	1.0480263670	0.4282717773
C	-6.3234121788	2.8733142522	-0.6353761035
H	-6.1666391306	3.4989817281	0.2482282315
H	-7.3989855069	2.6953754618	-0.7472195745
H	-5.9823754891	3.4337268533	-1.5112444731
C	-5.8017356847	0.5913932150	-1.6658682828
H	-5.2771781013	-0.3542283261	-1.5010753360

H	-5.4449299936	1.0184790483	-2.6084462557
H	-6.8706303423	0.3771904730	-1.7790630977
Si	-0.7565410939	-1.6375301196	2.2364543534
N	-0.3584712957	-2.5850544038	0.6813883311
N	0.5432204614	-4.0827190865	-0.6258354870
C	-0.2142072356	-2.8665271811	3.5832953427
H	0.8433468638	-3.1259794888	3.5501752823
H	-0.7860664724	-3.7974108767	3.4892558923
H	-0.4378693818	-2.4283091301	4.5642517675
C	-2.6565769210	-1.7609361645	2.3214490019
H	-3.2110005453	-0.9470200761	1.8450720628
H	-2.9552521992	-1.7800539209	3.3770505570
H	-2.9799169551	-2.7113402021	1.8793122681
C	0.6033273144	-3.5604131884	0.6512332703
C	-0.9986792953	-2.4773921154	-0.5601727154
C	-1.9976503793	-1.6189076420	-1.0277181918
H	-2.4102608818	-0.8444920965	-0.3923721153
C	-2.4340880288	-1.7775200458	-2.3431370995
H	-3.1964385969	-1.1101574552	-2.7344506387
C	-1.8904855273	-2.7699842925	-3.1733191330
H	-2.2504113890	-2.8723069118	-4.1941839717
C	-0.8841232605	-3.6218174595	-2.7167598488
H	-0.4664780560	-4.3784155565	-3.3723408626
C	-0.4350379424	-3.4557512202	-1.4026322345
C	1.4040192206	-5.2010776329	-1.0163685945
H	2.0544643161	-5.3246696812	-0.1454203933
C	0.5959993751	-6.4938944382	-1.2054758033
H	0.0307885468	-6.7228826195	-0.2973008128

H	1.2645528952	-7.3357522337	-1.4197390596
H	-0.1137737858	-6.4071454592	-2.0345060135
C	2.2789424765	-4.8735272290	-2.2348724452
H	2.7923576955	-3.9193612758	-2.0933635140
H	1.6949759307	-4.8051810760	-3.1574185004
H	3.0314773214	-5.6578567833	-2.3762757695

[Tism$^{Pr^iBenz}$]H (2:1 conformation)

atom	x	y	z
Si	2.4992053623	6.4131182842	6.8109258331
Si	2.9561294137	9.0623774273	5.0639236784
Si	0.6026938640	7.0361071059	4.2865119266
N	5.3980511549	5.7045263765	6.6733554535
C	2.3174064522	7.2760071730	5.1112935391
H	3.0136728505	6.7625596559	4.4305455245
N	3.8757321622	10.5242296864	2.6305251268
N	1.0461158216	4.6088233923	2.6047975866
N	3.8028912727	8.2799514943	2.4822086405
N	-0.5159678270	4.4692143183	4.2247910951
N	4.7148407866	6.8826511461	8.4695144228
C	6.5232175247	5.8894987445	7.4660645689
C	5.3221347403	4.9436536136	5.4158826822
H	4.2780324643	5.0154014625	5.1006452822
C	0.3996047093	5.2446596805	3.6649260672
C	4.3670070474	10.2042863978	1.3715925464
C	1.8425176231	4.6384383391	6.7966616937
H	2.3489677888	4.0053501113	6.0606988158

H	0.7734862068	4.5885347625	6.5703996600
H	2.0076874889	4.1936110584	7.7846367467
C	4.3349012410	6.3252165553	7.3305808826
C	-0.4886763562	3.2805474243	3.5224793731
C	7.8647576131	5.5002996517	7.3450149706
H	8.2298934991	4.9280221714	6.4998550949
C	0.4816901472	3.3454862299	2.4921216610
C	0.2983312297	8.1665134296	2.7915521092
H	1.0979668770	8.1255449801	2.0491017609
H	0.1779864053	9.2129480248	3.0898618163
H	-0.6369966860	7.8583013077	2.3098535248
C	4.3158975246	8.7912740098	1.3041567590
C	6.0681359497	6.6316580334	8.5838057619
C	3.5455072361	9.3265411689	3.2572005843
C	1.6583785814	7.3788761790	8.1951707679
H	2.0658073990	8.3887476613	8.2968995828
H	1.8340502857	6.8716474490	9.1482210638
H	0.5783779630	7.4515810272	8.0353432750
C	4.4897549769	9.4009194350	6.1207194541
H	5.3307942501	8.7645716631	5.8283588446
H	4.3132356053	9.2341380084	7.1868163456
H	4.8029723012	10.4438534875	5.9886419402
C	8.7319546809	5.8779179116	8.3647491935
H	9.7781373758	5.5923961137	8.2994151426
C	8.2883623055	6.6191840004	9.4783541410
H	9.0017644897	6.8917712004	10.2506907497
C	5.6338622318	3.4567973124	5.6431690328
H	5.4740694730	2.8957739841	4.7167508409

H	4.9808671808	3.0391512003	6.4148209499
H	6.6700639451	3.3021123818	5.9554216581
C	-0.8256365127	7.3026006115	5.4892642931
H	-0.8471391755	6.5469631588	6.2777414219
H	-1.7726061801	7.2233255183	4.9466699998
H	-0.7802100366	8.2943009773	5.9513416163
C	5.2644909902	10.2878035590	-0.8358526104
H	5.6374775644	10.8534590684	-1.6847150842
C	4.7531411798	8.1220574837	0.1529282952
H	4.7258550760	7.0378912092	0.1063938236
C	1.6150252382	10.2959732418	5.6028103769
H	0.8361627757	10.4518739187	4.8510947902
H	2.0337368516	11.2741308261	5.8610927554
H	1.1270306433	9.9146448216	6.5062520089
C	0.6928567563	2.2676553612	1.6203684731
H	1.4209420627	2.3037138238	0.8183428025
C	6.1844664953	5.5770579012	4.3140836304
H	6.1034763006	4.9883416595	3.3953347977
H	7.2393717446	5.6149154949	4.5966802011
H	5.8495393895	6.5932889689	4.0892487547
C	-1.2582202395	2.1228702357	3.7020665315
H	-1.9986315674	2.0813898510	4.4947874796
C	4.8400206549	10.9715548895	0.2981492121
H	4.8801951594	12.0541863659	0.3324070140
C	2.0979854195	5.1871847287	1.7517226484
H	2.3419217995	6.1597692281	2.1863010935
C	3.7611100622	11.8677490505	3.2214225899
H	3.3709731701	11.7017828474	4.2268068674

C	-0.0819580874	1.1294310522	1.8147875203
H	0.0574703262	0.2778012026	1.1548142777
C	6.9590842915	7.0015812876	9.6006658381
H	6.6024377801	7.5701438980	10.4537503379
C	-1.0438301165	1.0539764305	2.8423279543
H	-1.6260320634	0.1441896442	2.9568223420
C	3.3823549964	4.3473092282	1.7920247027
H	4.1910771393	4.8893973722	1.2926883980
H	3.2650965204	3.3821239726	1.2934165554
H	3.6863655578	4.1580715008	2.8251780402
C	2.7396681476	12.7305572567	2.4672639824
H	2.6064118436	13.6867256795	2.9832505524
H	1.7696386438	12.2274114046	2.4207629855
H	3.0605953608	12.9421908433	1.4439572679
C	5.2243892124	8.8815124706	-0.9094617980
H	5.5699063266	8.3882757758	-1.8132179013
C	1.5920090386	5.4204079936	0.3205807976
H	2.3591597009	5.9332547370	-0.2679677658
H	0.6944589622	6.0450513563	0.3257823860
H	1.3480357095	4.4795601160	-0.1817667204
C	5.1336927819	12.5410913714	3.3639098281
H	5.0270153536	13.4905016969	3.8981644630
H	5.5899647292	12.7510904908	2.3932754242
H	5.8196928225	11.9040251086	3.9295611196

[Tism$^{Pr^iBenz*}$]H (2:1 conformation)

atom	x	y	z
Si	1.9735187717	-0.5074003334	1.6939074936
N	2.7934941235	0.1519875490	0.2146471676
N	2.9299825587	0.6746356213	-1.8966685532
C	0.1907349251	0.1102071349	1.6518418216
C	2.8868628729	0.0774467898	3.2427558724
H	3.1177454630	1.1469678577	3.2292320424
H	3.8208011823	-0.4701161111	3.4035093414
H	2.2496706807	-0.1024279644	4.1155977507
C	2.2089555983	-2.3707858724	1.5184550351
H	1.6714048069	-2.7539549394	0.6471882625
H	1.8814801535	-2.9412310541	2.3917830406
H	3.2758815679	-2.5759225850	1.3698083507
C	2.0289523732	0.3983078550	-0.9071936298
C	4.1643333363	0.2588364123	-0.0811104056
C	5.3159253376	0.0915010586	0.6926288741
H	5.2574717550	-0.1698448554	1.7425691068
C	6.5528328804	0.2770660678	0.0759409602
H	7.4621273430	0.1529592681	0.6564367540
C	6.6394317257	0.6235223858	-1.2804156502
H	7.6154122603	0.7634873383	-1.7359051963
C	5.4942427384	0.7915669155	-2.0581896024
H	5.5764985276	1.0579731409	-3.1055344305
C	4.2535563743	0.6021108888	-1.4430925782
C	2.4668905837	0.9888168587	-3.2576669087

H	1.3791428646	0.9354700472	-3.1617189707
C	2.8478136638	2.4149402312	-3.6758338120
H	2.4879731880	3.1393882271	-2.9393010258
H	2.3921638524	2.6538897061	-4.6422990471
H	3.9297778159	2.5419759495	-3.7767492851
C	2.9156901269	-0.0747627218	-4.2684450082
H	2.5983556848	-1.0695063064	-3.9418918256
H	4.0019535153	-0.0902647447	-4.3981254957
H	2.4651798019	0.1256959263	-5.2460255825
Si	0.0008506786	1.9551859675	2.0121859878
N	0.1342787371	2.2683570415	3.7956983706
N	-0.5601324226	2.0230349778	5.8476859697
C	1.3053303582	2.9947732663	1.1285786949
H	2.3337928545	2.8116706679	1.4511993931
H	1.0874543608	4.0624589344	1.2382510890
H	1.2569238291	2.7450449203	0.0640007796
C	-1.6672088692	2.6278366698	1.4511412621
H	-2.4977146424	2.2254477931	2.0334197379
H	-1.8391483870	2.3839841421	0.3965998055
H	-1.6803351966	3.7193489358	1.5500067637
C	-0.7171610905	1.5309073726	4.5847118410
C	0.8208661554	3.2272000119	4.5634036623
C	1.7779396439	4.1955241615	4.2472365727
H	2.1342307943	4.3280113219	3.2340320138
C	2.2663545563	4.9941796327	5.2799245567
H	3.0103004767	5.7533865997	5.0568058214
C	1.8158161160	4.8352141715	6.5981450372
H	2.2145297815	5.4731182943	7.3813967533

C	0.8640904913	3.8701234771	6.9216422283
H	0.5239089349	3.7554895024	7.9440590873
C	0.3726962069	3.0673982360	5.8883712500
C	-1.3396169192	1.4817022684	6.9728770344
H	-1.9371769031	0.6952929594	6.5043837140
C	-0.4385595425	0.8361678524	8.0337723167
H	0.2091502517	0.0806579555	7.5792744929
H	-1.0536098318	0.3464187354	8.7954964142
H	0.1975209711	1.5687721952	8.5388365415
C	-2.2978421481	2.5313354237	7.5512898874
H	-2.9370420008	2.9397003890	6.7630440738
H	-1.7649080222	3.3626533085	8.0219196204
H	-2.9393596532	2.0721266079	8.3102435442
Si	-1.0823154891	-1.1090015740	2.3633088451
N	-1.2057855978	-2.4100829681	1.0992199276
N	-1.6436117255	-3.0537294150	-0.9352431188
C	-0.5623260889	-1.8975342680	3.9969712659
H	0.3955928853	-2.4230945875	3.9650543374
H	-1.3263421969	-2.5959473024	4.3546393785
H	-0.4888913488	-1.0833126428	4.7234030432
C	-2.8438406744	-0.4765794028	2.5332842711
H	-3.2128198761	-0.0745751223	1.5863113639
H	-2.8999529570	0.2851706472	3.3134510506
H	-3.4917769501	-1.3157103026	2.8137071220
C	-1.5350482778	-1.9392641122	-0.1524308267
C	-1.1214601428	-3.8111224084	1.1044168104
C	-0.8319728171	-4.7445244194	2.1034100210
H	-0.6213067555	-4.4337079800	3.1190333468

C	-0.8212563664	-6.0952985208	1.7572880646
H	-0.5986935849	-6.8382090614	2.5175298588
C	-1.0913829717	-6.5084139422	0.4445445832
H	-1.0744610717	-7.5668985551	0.2015213663
C	-1.3826483918	-5.5832846378	-0.5575601061
H	-1.5893013237	-5.9160452582	-1.5682483302
C	-1.3971584707	-4.2288504305	-0.2128935997
C	-1.9592909568	-2.9305331153	-2.3661974253
H	-2.0789744926	-1.8515750318	-2.4956798151
C	-0.7886828671	-3.3939393182	-3.2433909307
H	0.1226976479	-2.8522219534	-2.9731685362
H	-1.0086334185	-3.1952071993	-4.2975519571
H	-0.5930323804	-4.4654862917	-3.1386500651
C	-3.2878428639	-3.6112458066	-2.7198336108
H	-4.0919468240	-3.2340419017	-2.0811452466
H	-3.2416916053	-4.6979420521	-2.6021269853
H	-3.5490410991	-3.3994189583	-3.7618433835
H	-0.0427079496	0.0651688377	0.5717874778

[Tism$^{Pr^{i}Benz}$]H (3:0 conformation)

atom	x	y	z
H	0.0000000000	0.0000000000	0.2492599499
Si	-0.8660655078	-1.6402640951	1.7371057533
N	0.5074819998	-2.3843788808	-0.6073603085
N	-0.6582214667	-4.1568534953	0.1448361216
C	0.0000000000	0.0000000000	1.3516772421
C	-2.7599770773	-1.5980294468	1.6342288506

H	-3.1948090419	-0.8728425169	2.3287128117
H	-3.1874798067	-2.5751757463	1.8848270247
H	-3.0759675532	-1.3201159804	0.6269906115
C	-0.4425040187	-2.2875922915	3.4758719919
H	-0.9704114393	-3.2197987698	3.7052008193
H	-0.7613605611	-1.5492271163	4.2214848797
H	0.6251285293	-2.4678723378	3.6277731508
C	-0.2765339724	-2.8309192608	0.3595564690
C	0.6545988872	-3.4273393465	-1.4983007423
C	1.3779630104	-3.4743555120	-2.6976729255
H	1.9264119667	-2.5996415855	-3.0325921746
C	1.3569454482	-4.6544731609	-3.4291493767
H	1.9042234723	-4.7204774878	-4.3648899886
C	0.6280101317	-5.7738092270	-2.9776301997
H	0.6264730661	-6.6818224760	-3.5743019675
C	-0.0950592500	-5.7478120873	-1.7890017645
H	-0.6508467955	-6.6214721555	-1.4670802812
C	-0.0731011434	-4.5565746111	-1.0498107218
C	-1.5410649747	-4.9529330424	1.0095671002
H	-1.7779346705	-4.3002705585	1.8524337668
C	-0.8221338502	-6.1857140891	1.5755930181
H	0.0940501710	-5.8919784190	2.0958849238
H	-0.5527772098	-6.8977529941	0.7913682495
H	-1.4737473705	-6.7010533704	2.2888351533
C	-2.8597529540	-5.2978362829	0.3022030632
H	-3.3564958736	-4.3889737615	-0.0483296423
H	-3.5333645492	-5.8145054277	0.9938394083
H	-2.6974732474	-5.9489033954	-0.5607898593

Si	-0.9874776213	1.5701667786	1.7371057533
N	-2.3186736829	0.7526971367	-0.6073603085
N	-3.2708299934	2.6484632591	0.1448361216
C	-0.0039455582	3.1892249862	1.6342288506
H	0.8415007278	3.2032070490	2.3287128117
H	-0.6364277122	4.0480263598	1.8848270247
H	0.3947298016	3.3239240325	0.6269906115
C	-1.7598610286	1.5270158673	3.4758719919
H	-2.3032218101	2.4503003434	3.7052008193
H	-0.9609897584	1.4339711455	4.2214848797
H	-2.4498044025	0.6925589819	3.6277731508
C	-2.3133810097	1.6549450755	0.3595564690
C	-3.2954623851	1.1467704077	-1.4983007423
C	-3.6978616404	0.5438267835	-2.6976729255
H	-3.2145616372	-0.3685009086	-3.0325921746
C	-4.7093647226	1.1520873508	-3.4291493767
H	-5.0401651586	0.7111328424	-4.3648899886
C	-5.3142705331	2.3430318856	-2.9776301997
H	-6.0998645408	2.7983696480	-3.5743019675
C	-4.9302216588	2.9562297690	-1.7890017645
H	-5.4089396994	3.8743859366	-1.4670802812
C	-3.9095587958	2.3415947528	-1.0498107218
C	-3.5188333507	3.8110679382	1.0095671002
H	-2.8351762115	3.6898718702	1.8524337668
C	-4.9459186166	3.8048458441	1.5755930181
H	-5.1496280749	2.8645393722	2.0958849238
H	-5.6972407171	3.9275956033	0.7913682495
H	-5.0664087656	4.6268293468	2.2888351533

C	-3.1581843291	5.1255368482	0.3022030632
H	-2.1227148372	5.1012975750	-0.0483296423
H	-3.2688271362	5.9672361743	0.9938394083
H	-3.8031648414	5.3105320560	-0.5607898593
Si	1.8535431291	0.0700973164	1.7371057533
N	1.8111916832	1.6316817442	-0.6073603085
N	3.9290514601	1.5083902362	0.1448361216
C	2.7639226356	-1.5911955394	1.6342288506
H	2.3533083141	-2.3303645321	2.3287128117
H	3.8239075188	-1.4728506135	1.8848270247
H	2.6812377516	-2.0038080521	0.6269906115
C	2.2023650473	0.7605764242	3.4758719919
H	3.2736332493	0.7694984264	3.7052008193
H	1.7223503195	0.1152559708	4.2214848797
H	1.8246758732	1.7753133559	3.6277731508
C	2.5899149821	1.1759741853	0.3595564690
C	2.6408634979	2.2805689389	-1.4983007423
C	2.3198986300	2.9305287285	-2.6976729255
H	1.2881496704	2.9681424941	-3.0325921746
C	3.3524192745	3.5023858101	-3.4291493767
H	3.1359416863	4.0093446454	-4.3648899886
C	4.6862604013	3.4307773414	-2.9776301997
H	5.4733914748	3.8834528280	-3.5743019675
C	5.0252809088	2.7915823183	-1.7890017645
H	6.0597864949	2.7470862189	-1.4670802812
C	3.9826599391	2.2149798584	-1.0498107218
C	5.0598983253	1.1418651043	1.0095671002
H	4.6131108821	0.6103986883	1.8524337668

C	5.7680524667	2.3808682450	1.5755930181
H	5.0555779039	3.0274390468	2.0958849238
H	6.2500179268	2.9701573908	0.7913682495
H	6.5401561361	2.0742240236	2.2888351533
C	6.0179372831	0.1722994347	0.3022030632
H	5.4792107108	-0.7123238135	-0.0483296423
H	6.8021916854	-0.1527307466	0.9938394083
H	6.5006380888	0.6383713394	-0.5607898593

[Tism$^{Pr^iBenz*}$]H (3:0 conformation)

**atom**	**x**	**y**	**z**
H	0.0255221527	0.0034818278	0.4584100931
Si	1.7823279202	-0.5176962074	1.9571819968
N	2.8507180931	0.2015473233	0.6815601233
N	3.3663035807	1.0875544568	-1.2417364468
C	0.0169639567	0.0162463811	1.5483017465
C	2.3597214681	0.0880815000	3.6564319914
H	2.3300187000	1.1762191085	3.7593584825
H	3.3840177959	-0.2344298769	3.8705735402
H	1.7167715403	-0.3389212595	4.4356029743
C	2.1315468620	-2.3698470969	1.8632287072
H	1.7499510534	-2.7606929859	0.9170943829
H	1.6741564914	-2.9338970669	2.6813120017
H	3.2107985296	-2.5496146522	1.9029976812
C	2.3120756544	0.8668950856	-0.4011398267
C	4.2344084033	0.0082908448	0.5100100691
C	5.2122847761	-0.5906736939	1.3081818227

H	4.9701122976	-1.0185198739	2.2746601198
C	6.5220738997	-0.6219740555	0.8297144396
H	7.2990501027	-1.0846119796	1.4309301130
C	6.8493583296	-0.0643697112	-0.4147093699
H	7.8765521101	-0.1037212026	-0.7654102615
C	5.8796749826	0.5435668137	-1.2119415554
H	6.1475993967	0.9730139964	-2.1703176685
C	4.5670316200	0.5756376018	-0.7337136087
C	3.1566384646	1.7684009014	-2.5291446155
H	2.0879259171	1.9978367657	-2.5095288379
C	3.9354511983	3.0872549316	-2.6140456640
H	3.6962877940	3.7289913892	-1.7610097929
H	3.6645122059	3.6208755301	-3.5309312763
H	5.0179995786	2.9294249900	-2.6281285886
C	3.4267976751	0.8317703918	-3.7141999762
H	2.8278044165	-0.0789648260	-3.6220212912
H	4.4799596614	0.5422671885	-3.7808103170
H	3.1565770996	1.3280851208	-4.6521637016
Si	-0.4099440638	1.8173643012	1.9187110109
N	-1.5994634290	2.3284931905	0.6494973795
N	-2.6889800433	2.2468406482	-1.2362650638
C	-1.1876559524	2.0588996048	3.6289308693
H	-2.0970330231	1.4711369307	3.7798466434
H	-1.4414406889	3.1088544524	3.8085331023
H	-0.4673813940	1.7642201808	4.4017328476
C	1.0066165713	3.0531268947	1.7639762788
H	1.5082449830	2.9074212445	0.8045038353
H	1.7496994056	2.9474429231	2.5601183677

H	0.6204425349	4.0769550459	1.8065493574
C	-1.9245558148	1.4880868137	-0.3961578777
C	-2.1588662727	3.6048259780	0.4547588957
C	-2.1269762827	4.7774318225	1.2130967268
H	-1.6006417838	4.8261239422	2.1599344816
C	-2.7988531312	5.8951854930	0.7195839890
H	-2.7866063835	6.8190518320	1.2905222185
C	-3.4891777957	5.8453177857	-0.4996402460
H	-4.0021640443	6.7319364036	-0.8606770959
C	-3.5310758241	4.6757659332	-1.2577657668
H	-4.0701333546	4.6500058213	-2.1975971537
C	-2.8589141977	3.5544020886	-0.7645385459
C	-3.2162708257	1.6721533847	-2.4836547169
H	-2.8541756673	0.6412446193	-2.4471699135
C	-4.7502818547	1.6485105452	-2.4976470085
H	-5.1335096417	1.1347013587	-1.6111530725
H	-5.1068400363	1.1140843457	-3.3842150047
H	-5.1800133183	2.6544307849	-2.5194467882
C	-2.6122272414	2.3526153111	-3.7192032155
H	-1.5197604244	2.3185611543	-3.6751947011
H	-2.9174684234	3.3998314958	-3.8052891212
H	-2.9367219140	1.8342935973	-4.6275562514
Si	-1.3302156501	-1.2509305467	1.9376262195
N	-1.2188636249	-2.5111955518	0.6386326397
N	-0.6629226983	-3.3593020105	-1.2906020973
C	-1.1145126467	-2.0748184897	3.6302038028
H	-0.1546177629	-2.5823748600	3.7532353549
H	-1.9027589170	-2.8126137422	3.8140692038

H	-1.1945345228	-1.3153033164	4.4175712725
C	-3.1134405672	-0.6378701935	1.8607642198
H	-3.2883287392	-0.1417934623	0.9039412779
H	-3.3508126097	0.0671187300	2.6631034480
H	-3.8057284466	-1.4814522808	1.9500441819
C	-0.3425146191	-2.3593466588	-0.4173555786
C	-2.0784432769	-3.6030420206	0.4184429326
C	-3.1071796745	-4.1650737795	1.1778127568
H	-3.3780206494	-3.7654104952	2.1487947533
C	-3.7808317796	-5.2679003813	0.6540550236
H	-4.5860087121	-5.7208198348	1.2251369531
C	-3.4350446059	-5.8010606055	-0.5956310776
H	-3.9787358588	-6.6589444325	-0.9802681188
C	-2.4020418971	-5.2513521715	-1.3538818159
H	-2.1420824548	-5.6753478975	-2.3167400740
C	-1.7248205357	-4.1472202957	-0.8298250258
C	0.0603116446	-3.4913206147	-2.5647352343
H	0.7979896600	-2.6864074480	-2.5084891707
C	0.8054515718	-4.8284704272	-2.6645928977
H	1.4619239179	-4.9663720479	-1.8004509678
H	1.4218295848	-4.8444298654	-3.5693514379
H	0.1220602170	-5.6815386573	-2.7120187995
C	-0.8582572717	-3.2213453358	-3.7640127834
H	-1.3413858914	-2.2455059468	-3.6591053476
H	-1.6403245175	-3.9800355951	-3.8645797472
H	-0.2739980692	-3.2185907488	-4.6901081988

REFERENCES

(1) (a) McNally, J. P.; Leong, V. S.; Cooper, N. J. in *Experimental Organometallic Chemistry*, Wayda, A. L.; Darensbourg, M. Y., Eds.; American Chemical Society: Washington, DC, 1987; Chapter 2, pp 6-23.
(b) Burger, B.J.; Bercaw, J. E. in *Experimental Organometallic Chemistry*; Wayda, A. L.; Darensbourg, M. Y., Eds.; American Chemical Society: Washington, DC, 1987; Chapter 4, pp 79-98.
(c) Shriver, D. F.; Drezdzon, M. A.; *The Manipulation of Air-Sensitive Compounds*, 2nd Edition; Wiley-Interscience: New York, 1986.

(2) (a) Gottlieb, H. E.; Kotlyar, V.; Nudelman, A. *J. Org. Chem.* **1997**, *62*, 7512-7515.
(b) Fulmer, G. R.; Miller, A. J. M.; Sherden, N. H.; Gottlieb, H. E.; Nudelman, A.; Stoltz, B. M.; Bercaw, J. E.; Goldberg, K. I. *Organometallics* **2010**, *29*, 2176-2179.

(3) (a) Harris, R. K.; Becker, E. D.; De Menezes, S. M. C.; Goodfellow, R.; Granger, P. *Pure Appl. Chem.* **2001**, *73*, 1795-1818.
(b) Harris, R. K.; Becker, E. D.; De Menezes, S. M. C.; Granger, P.; Hoffman, R. E.; Zilm, K. W. *Pure Appl. Chem.* **2008**, *80*, 59-84.

(4) Starikova, O. V.; Dolgushin, G. V.; Larina, L. I.; Ushakov, P. E.; Komarova, T. N.; Lopyrev, V. A. *Russ. J. Org. Chem.* **2003**, *39*, 1467-1470.

(5) (a) Qi, G.; Nitto, Y.; Saiki, A.; Tomohiro, T.; Nakayama, Y.; Yasuda, H. *Tetrahedron* **2003**, *59*, 10409-10418.
(b) Sakurai, H.; Tominaga, K.; Watanabe, T.; Kumada, M. *Tetrahedron Lett.* **1966**, 5493-5497.

(6) Yousef, R. I.; Walfort, B.; Ruffer, T.; Wagner, C.; Schmidt, H.; Herzog, R.; Steinborn, D. *J. Organomet. Chem.* **2005**, *690*, 1178-1191.

(7) Schmidbaur, H.; Adlkofer, J.; Schwirten, K. *Chem. Ber*. **1972**, 105, 3382-3388.

(8) (a) Sheldrick, G. M. SHELXTL, An Integrated System for Solving, Refining, and Displaying Crystal Structures from Diffraction Data; University of Göttingen,

Göttingen, Federal Republic of Germany, 1981.

(b) Sheldrick, G. M. *Acta Cryst.* **2008**, *A64*, 112-122.

(c) Sheldrick, G. M. *Acta Cryst.* **2015**, *A71*, 3-8.

(9) Jaguar 7.7, Schrödinger, LLC, New York, NY 2010.

(10) (a) Becke, A. D. *J. Chem. Phys.* **1993**, *98*, 5648-5652.

(b) Becke, A. D. *Phys. Rev. A* **1988**, *38*, 3098-3100.

(c) Lee, C. T.; Yang, W. T.; Parr, R. G. *Phys. Rev. B* **1988**, *37*, 785-789.

(d) Vosko, S. H.; Wilk, L.; Nusair, M. *Can. J. Phys.* **1980**, *58*, 1200-1211.

(e) Slater, J. C. *Quantum Theory of Molecules and Solids, Vol. 4: The Self-Consistent Field for Molecules and Solids*; McGraw-Hill: New York, 1974.

(11) (a) Hay, P. J.; Wadt, W. R. *J. Chem. Phys.* **1985**, *82*, 270-283.

(b) Wadt, W. R.; Hay, P. J. *J. Chem. Phys.* **1985**, *82*, 284-298.

(c) Hay, P. J.; Wadt, W. R. *J. Chem. Phys.* **1985**, *82*, 299-310.
